# Study on Molten Pool Dynamic Behavior of Laser–MIG Hybrid Welding for 10CrNiCu Steel Under Different Assembly Conditions

**DOI:** 10.3390/ma19183863

**Published:** 2026-09-10

**Authors:** Mingzhu Qian, Wenyong Zhao, Hui Liu, Dejun Yan, Guoxiang Xu, Wen Liu, Qingxian Hu

**Affiliations:** 1 School of Materials Science and Engineering, Jiangsu University of Science and Technology, Zhenjiang 212003, China; 2 School of Materials Science and Hydrogen Energy, Foshan University, Foshan 528051, China

**Keywords:** laser–MIG hybrid welding, molten pool dynamics, 10CrNiCu steel, assembly gap, assembly misalignment

## Abstract

Laser–MIG hybrid welding is increasingly used in shipbuilding for medium-thick steel plates, where assembly-induced gaps and misalignment often compromise weld quality. In this study, a three-dimensional transient numerical model is established to simulate the molten pool dynamics during full-penetration laser–MIG hybrid welding of 10CrNiCu steel under varying gaps and misalignment conditions. The model incorporates coupled heat transfer, fluid flow, keyhole behavior, and droplet transfer and is validated against experimental weld profiles. The simulation results reveal that increasing gap size broadens the heat distribution, reduces keyhole depth, and decreases the bridging capacity of the filler metal. Larger misalignment enhances the step effect, promotes gravity-driven downward flow of liquid metal, and increases keyhole instability and porosity risk. The adaptability to assembly errors is further assessed. As welding current increases, the gap tolerance slightly improves from 1.61 mm to 1.65 mm, whereas the misalignment tolerance markedly decreases from 3.00 mm to 1.82 mm due to the earlier onset of porosity defects. These findings provide quantitative guidance for optimizing welding parameters to accommodate realistic assembly variations in marine steel fabrication.

## 1. Introduction

10CrNiCu steel is widely used in the manufacturing of key structures in the field of shipbuilding and marine engineering due to its excellent corrosion resistance, high strength, and low temperature toughness [1,2]. However, its manufacturing currently relies mainly on conventional welding methods, which result in problems such as high residual stress, significant deformation, and grain coarsening in the welded joints. Laser–arc hybrid welding (LAHW), as an advanced and high efficiency welding technique, combines the advantages of laser welding, including high energy density and large penetration efficiency, and those of arc welding, such as excellent bridging capability and strong gap adaptability. Their synergistic interaction can significantly suppress defects prone to occur in laser welding, including porosity and cracking. Furthermore, it can better meet the welding requirements of 10CrNiCu steel while achieving high efficiency and high quality welding [3].

The research and application of LAHW in the field of marine engineering metal materials has gradually formed a research system with AH36, X80, EH36 series of typical high-strength ship plates and conventional marine engineering structural steel as the core [4,5,6]. Churiaque et al. [7] had obtained full penetration T-joints of 8 mm thick EH36 steel plate by LAHW and using 2F position and 0 gap with square groove, and the influence of key processing parameters such as laser power, welding speed, and wire feeding speed on formation quality has been investigated. The results demonstrate that increasing the welding rate within an appropriate range contributes to a better synergistic effect between the laser and arc heat sources, thus ensuring full metallurgical bonding between the flange and web. In order to further meet the industrial application requirements of medium and heavy plates, Song et al. [8] realized the laser–arc hybrid welding of 25 mm thick EH36 steel plate by reducing the groove diameter and found that when the heat input of the molten pool was large, increasing the welding speed would lead to a significant temperature gradient between the molten pool center and the sidewall. In addition, the increase in groove width reduced the rate of hardness change to a certain extent. However, in the actual manufacturing of large ships and offshore engineering equipment, welding often involves seams that extend for kilometers, making assembly errors such as variations in welding gap and misalignment practically unavoidable. In response to these challenges, researchers have conducted extensive studies focusing on assembly deviations [9,10]. Regarding laser welding heat sources, to explore the influence of assembly gaps on the tensile properties of steel joints fabricated from steels with different strength grades, Lee et al. [11] applied laser welding to join ultra-high-strength steels with different base metal grades under various gap conditions. Their results demonstrated that when the gap width exceeded 0.3 mm, the incomplete penetration depth at the weld root increased with rising gap width, regardless of the base metal grade, for all welds produced without filler wire. For arc welding heat sources, Li et al. [12] fabricated 6061 aluminum alloy joints with varying gap widths via CMT (Cold Metal Transfer) welding and characterized the weld bead morphology of the joints under different gap assembly conditions. Their results showed that a narrow gap could be fully filled by the molten metal, resulting in a large weld bead width. As the gap width increased, the amount of filler metal required to fill the gap rose, which in turn reduced the weld bead width, deteriorated formation uniformity and caused slight ripples on the weld surface. The aforementioned studies have conducted systematic investigations on assembly errors under conventional welding heat sources.

The heat and mass transfer behavior in the molten pool is closely related to weld formation as well as the microstructure and properties of the weld, and the numerical simulation of the welding process provides an effective means for investigating the heat transfer and fluid flow behavior in the molten pool [13]. Jing et al. [14] carried out targeted research on the melt flow behavior within the molten pool during narrow-gap laser welding of 316LN stainless steel. These works revealed that the assembly gap alters the heat input into the molten pool, weakens keyhole stability, and further disrupts the dynamic stability of the molten pool. Hao et al. [15] simulated the TIG welding process with preset assembly gaps using a CFD (Computational Fluid Dynamics) model. Their results showed that the amount of molten metal rises with increasing heat input, eventually forming a liquid bridge between the two sides of the workpiece. For plasma–MIG hybrid welding, Wu et al. [16] observed that the collapse of the plasma keyhole bottom leads to liquid bridge formation between the front and rear keyhole walls. Chen et al. [17] further pointed out that liquid bridge generation in pulsed laser beam welding is attributed to keyhole closure. These findings confirm that molten pool flow characteristics under assembly gaps vary with welding processes. Focusing on vertical gaps, Tang et al. [18] conducted numerical simulation of PLBW (pulsed laser beam welding) for gapped plates via a CFD model and found that vertical gaps affect the distribution and absorption of laser energy. To further clarify the influence of vertical gaps on weld formation, Yang et al. [19] developed a 3D CFD model considering both vertical and horizontal gaps and investigated laser–MIG hybrid welding of 6A01 aluminum alloy bottom lock joints with assembly gaps. Their analysis of vertical gap flow dynamics revealed that weld melt width and penetration depth increase with the rise in vertical gap size. The above studies on molten pool flow behavior under assembly gaps have provided theoretical support for improving the gap bridging capability of aluminum alloys. To enhance the gap bridging penetration performance of aluminum alloy laser welding, Zhou et al. [20] adopted alternating magnetic field-assisted laser welding for 5A06 aluminum alloy to mitigate insufficient heat input during gap bridging. They established a magnetic field-assisted laser welding model considering assembly gaps to simulate molten pool dynamics and MHD (Magnetohydrodynamics) during welding. The results showed that the magnetic field improves heat exchange between the molten pool and the heat source. As magnetic field frequency increases, lack of fusion defects are gradually eliminated, and full weld penetration can be achieved. Thus, heat transfer and molten pool dynamics are the core factors that determine the microstructure, service performance, and forming characteristics of welds.

In conclusion, researchers have conducted a considerable body of research on molten pool flow behavior of steel under assembly error conditions. Significant research gaps remain. Specifically, the dynamic behavior of the molten pool during laser–MIG hybrid welding of 10CrNiCu steel under different assembly conditions has not been systematically understood. Previous numerical studies on laser–arc hybrid welding have been relatively abundant for gap conditions, but those concerning misalignment are still scarce. Moreover, most of the existing literature treats gap and misalignment separately, lacking comparative analyses of their respective effects on heat transfer, fluid flow, and keyhole behavior. Meanwhile, the tolerance of 10CrNiCu steel under varying welding currents has not been addressed in the published work. This study attempts to fill the above gaps from three aspects. First, unlike previous CFD studies that mainly adopted simplified heat sources or two-dimensional models, this work establishes a unified three-dimensional numerical model that enables a comparative analysis of the influences of gap and misalignment on molten pool dynamics. Compared with existing simulation studies, the present model achieves simultaneous coupled computations of the “droplet–keyhole–molten pool” multiphysics under gap and misalignment conditions, thereby revealing the distinct effects of the two types of deviation on melt flow. Second, quantitative tolerance limits of 10CrNiCu steel under different welding currents are obtained, which can serve as a reference for parameter selection. Third, the simulated temperature fields, flow fields, and keyhole morphologies are analyzed in detail to elucidate the different mechanisms by which gap and misalignment affect molten pool behavior. The gap primarily alters the flow space and thermal distribution, whereas misalignment induces directional flow and pool asymmetry. This work provides practical guidance for selecting process parameters that accommodate actual assembly variations in shipbuilding manufacturing.

## 2. Experimental Description

**Figure 1:**
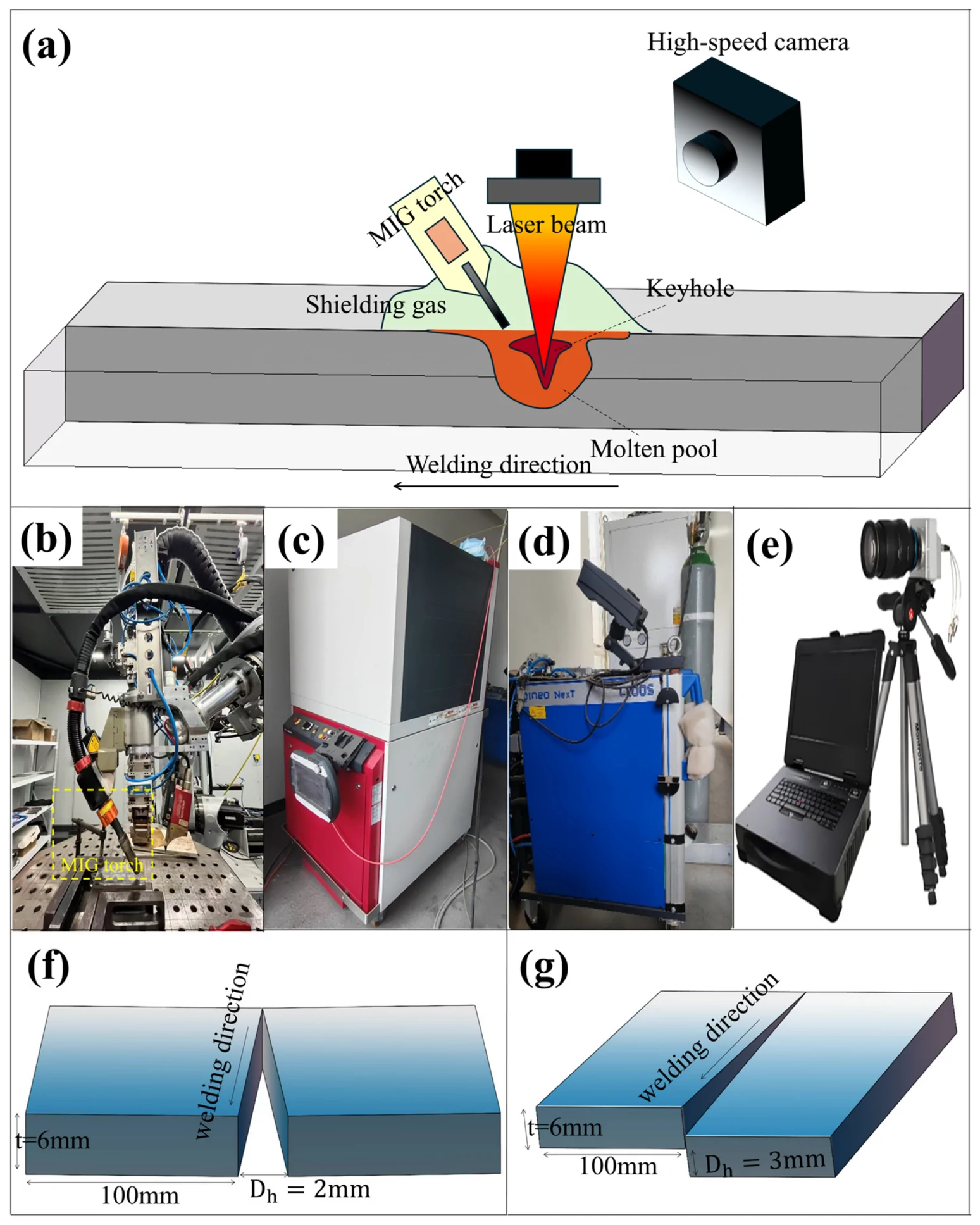
Experimental setup: (**a**) schematic diagram of laser and arc heat source configuration; (**b**) laser–arc hybrid welding equipment; (**c**) laser device; (**d**) MIG welding machine; (**e**) high-speed camera system; (**f**) schematic diagram of variable gap test; (**g**) schematic diagram of variable misalignment test.

The laser–arc hybrid welding configuration and equipment for 10CrNiCu steel is shown in Figure 1, where the arc is a leading heat source, as illustrated in Figure 1a,b. The fiber laser source used was Laserline LDF 11000-40 produced by Germany (Laserline GmbH, Mülheim-Kärlich, Germany), as present in Figure 1c, with a wavelength range of 900–1070 nm. The motion system employed was a CLOOS heavy-duty robot (CLOOS, Siegen, Germany), and the arc welding machine was CLOOS Qineo Next 452 (CLOOS, Siegen, Germany), as present in Figure 1d. A high-speed photography system, Optronis CP80-3-M-540 (Optronis GmbH, Kehl, Germany), was installed perpendicular to the welding direction to capture the droplet and molten pool morphology. In hybrid welding, the laser was perpendicular to the test plate, and the angle between the welding torch and the workpiece is 60°.

The hot-rolled 10CrNiCu steel plate specimens used in the experiments, manufactured in accordance with Chinese military standard GJB 5347-2004 [21], had dimensions of 150 mm × 100 mm × 6 mm, and the main chemical compositions are listed in Table 1. The welding wire utilized matched the base metal in chemical composition and had a diameter of 1.2 mm. In accordance with actual production conditions, the two tolerance experiments were conducted as described below. For the gap tolerance experiment, a 0 mm gap was fixed at one end of the joint, while a 2 mm feeler gauge was inserted at the opposite end to replicate the maximum gap condition. Tack welding was applied at the 0 mm gap end to maintain a stable gap throughout the welding process, as illustrated in Figure 1f. For the misalignment tolerance experiment, zero misalignment was set at one end of the joint, and a 3 mm feeler gauge was placed beneath one specimen at the other end to simulate the maximum misalignment scenario. Likewise, spot welding was performed at the 0 mm misalignment end to prevent any variation in misalignment during welding, and the corresponding experimental setup is presented in Figure 1g. In the preliminary exploratory experiments, the optimal ranges of the process parameters were determined through a series of tests: laser power of 4–4.5 kW, welding current of 230–250 A, welding speed of 18–20 mm/s, laser-to-wire distance of 2–3 mm, and defocus amount of 0 mm. To independently evaluate the effect of welding current on weld quality under different assembly errors, the subsequent experiments were carried out with the laser power fixed at 4 kW, the welding speed at 20 mm/s, the laser-to-wire distance at 2 mm, and the defocus amount at 0 mm, while the shielding gas composition was maintained at 82% Ar + 18% CO_2_. Only the welding current was varied within the range of 210 A to 250 A.

## 3. Mathematical Modeling

### 3.1. Basic Assumptions

Flow and heat transfer in the welding molten pool are dominated by the coupled action of multiple mechanical and thermophysical effects, namely gravity, arc force, laser-induced vapor recoil pressure, electromagnetic force, and surface tension, rendering the underlying mechanism highly complex. However, to emphasize the core research focus while balancing the feasibility and computational efficiency of numerical simulation, it is essential to simplify the actual physical processes prior to developing a molten pool numerical model [22,23]. Accordingly, the following assumptions are adopted in this work for the establishment of the molten pool flow and heat transfer model.

(1) The molten metal in the molten pool is treated as a laminar, incompressible Newtonian fluid.

(2) The internal pressure of the welding keyhole is assumed to communicate with the external environment, equivalent to atmospheric pressure.

(3) The thermophysical parameters of the material, including specific heat capacity and thermal conductivity, are considered to be solely temperature dependent.

(4) The enthalpy-porosity method is adopted to simulate the melting and solidification of molten metal during welding for a better description of thermophysical processes in the phase transformation zone.

It should be noted that the assumptions of laminar and incompressible Newtonian flow introduce certain limitations. The Reynolds number in the molten pool, estimated from the simulated peak velocity (~1.5 m/s) and pool width (~4 mm), is approximately 6500, which falls in the transition regime. However, the strong Marangoni convection, high temperature gradient, and the highly directional nature of the flow tend to suppress full turbulence development. Moreover, the laminar assumption has been widely adopted in previous CFD studies of laser–arc hybrid welding and has yielded acceptable agreement with experiments [22,23]. The incompressible Newtonian assumption is justified by the low Mach number (Ma << 1) and the linear shear–strain relationship of liquid metals. Nevertheless, the model does not capture local turbulent fluctuations, and its accuracy may be reduced under extremely high current or high-speed conditions, where flow becomes more intense.

### 3.2. Governing Equations

In this study, the enthalpy-porosity method proposed by Voller and Prakash [24] is adopted to handle the melting and solidification of the molten pool. The liquid fraction is defined as a linear function of temperature, and a Darcy-like momentum source term is employed to suppress the fluid velocity in the solid region. The governing equations for mass, momentum, and energy conservation are given as follows.

(1) Mass continuity equation.

(1) $$\nabla \cdot (\mathrm{\rho v})+\frac{\partial \rho }{\partial t}=0$$

where *ρ* is the density, *v* is the velocity vector, and *t* is the time.

(2) Momentum conservation equation.

Momentum equation in *x*-axis direction.

(2) $$\begin{array}{l} \rho \left[\frac{\partial v_{x}}{\partial t}+\left(v_{x}-v_{0}\right)\frac{\partial v_{x}}{\partial x}+v_{y}\frac{\partial v_{x}}{\partial y}+v_{z}\frac{\partial v_{x}}{\partial z}\right]=-\frac{\partial p}{\partial x}\\ +\mu \left(\frac{\partial^{2}v_{x}}{\partial x^{2}}+\frac{\partial^{2}v_{x}}{\partial y^{2}}+\frac{\partial^{2}v_{x}}{\partial z^{2}}\right)+g_{x} \end{array}$$

Momentum equation in the *y*-axis direction.

(3) $$\begin{array}{l} \rho \left[\frac{\partial v_{y}}{\partial t}+\left(v_{x}-v_{0}\right)\frac{\partial v_{y}}{\partial x}+v_{y}\frac{\partial v_{y}}{\partial y}+v_{z}\frac{\partial v_{y}}{\partial z}\right]=-\frac{\partial p}{\partial x}\\ +\mu \left(\frac{\partial^{2}v_{y}}{\partial x^{2}}+\frac{\partial^{2}v_{y}}{\partial y^{2}}+\frac{\partial^{2}v_{y}}{\partial z^{2}}\right)+g_{y} \end{array}$$

Momentum equation in the *z*-axis direction.

(4) $$\begin{array}{l} \rho \left[\frac{\partial v_{z}}{\partial t}+\left(v_{x}-v_{0}\right)\frac{\partial v_{z}}{\partial x}+v_{y}\frac{\partial v_{z}}{\partial y}+v_{z}\frac{\partial v_{z}}{\partial z}\right]=-\frac{\partial p}{\partial x}\\ +\mu \left(\frac{\partial^{2}v_{z}}{\partial x^{2}}+\frac{\partial^{2}v_{z}}{\partial y^{2}}+\frac{\partial^{2}v_{z}}{\partial z^{2}}\right)+g_{z} \end{array}$$

where *μ* is viscosity; *p* is pressure; *g_x_*, *g_y_*, and *g_z_* represent the momentum source terms in the *x*, *y*, and *z* directions, respectively, with the expression as follows.

(5) $$g_{x}=-\frac{{\left(1-f_{l}\right)}^{2}}{f_{l}^{3}+\varepsilon_{d}}A_{\mathrm{mush}}v_{x}+F_{x}$$

(6) $$g_{y}=-\frac{{\left(1-f_{l}\right)}^{2}}{f_{l}^{3}+\varepsilon_{d}}A_{\mathrm{mush}}v_{y}+F_{y}$$

(7) $$g_{z}=-\frac{{\left(1-f_{l}\right)}^{2}}{f_{l}^{3}+\varepsilon_{d}}A_{\mathrm{mush}}v_{z}+F_{z}$$

where *F_x_*, *F_y_*, and *F_z_* are the components of volume force inside the molten pool in the x, y, and z directions, respectively. The *ε_d_* is a small constant that ensures the denominator is not equal to 0; *A_mush_* is a constant. *f_l_* is the liquid volume fraction, and its expression is as follows.

(8) $$f_{l}=\left\{\begin{array}{cc} 0 & T\leq T_{s}\\ \frac{T-T_{s}}{T_{l}-T_{s}} & T_{s}<T\leq T_{l}\\ 1 & T>T_{l} \end{array}\right.$$

where *T* is the characterization temperature, *T_s_* is the solidus temperature, and *T_l_* is the liquidus temperature.

The volume source term drives the molten pool flow. The volume force includes electromagnetic force [25] and buoyancy force [26,27], and its components are shown in Equations (9)–(11).

(9) $$F_{x}=-\frac{\mu_{0}I^{2}}{4\pi^{2}\sigma_{j}^{2}r}\exp\left(-\frac{r^{2}}{2\sigma_{j}^{2}}\right)\left[1-\exp\left(-\frac{r^{2}}{2\sigma_{j}^{2}}\right)\right]{\left(1-\frac{z}{L}\right)}^{2}\frac{x}{r}$$

(10) $$F_{y}=-\frac{\mu_{0}I^{2}}{4\pi^{2}\sigma_{j}^{2}r}\exp\left(-\frac{r^{2}}{2\sigma_{j}^{2}}\right)\left[1-\exp\left(-\frac{r^{2}}{2\sigma_{j}^{2}}\right)\right]{\left(1-\frac{z}{L}\right)}^{2}\frac{y}{r}$$

(11) $$F_{z}=-\frac{\mu_{0}I^{2}}{4\pi^{2}\sigma_{j}^{2}r}{\left[1-\exp\left(-\frac{r^{2}}{2\sigma_{j}^{2}}\right)\right]}^{2}\left(1-\frac{z}{L}\right)+\mathrm{\rho g}\beta_{0}\Delta \left(T-T_{0}\right)$$

where *μ*_0_ is the magnetic permeability, $\sigma_{j}$ is the distribution radius of electromagnetic force, *g* is acceleration of gravity, *L* is the thickness of the workpiece, *β*_0_ is the thermal expansion coefficient, $r=\sqrt{{\left(x-\mu_{0}t\right)}^{2}+y^{2}}$, and *T*_0_ is the ambient temperature. The second term of right-hand side of Equation (11) is the buoyancy force.

(3) Energy conservation equation.

(12) $$\begin{array}{l} \frac{\partial \rho H_{\mathrm{ens}}}{\partial t}+\left(v_{x}-u_{0}\right)\frac{\partial \rho H_{\mathrm{ens}}}{\partial x}+v_{y}\frac{\partial \rho H_{\mathrm{ens}}}{\partial y}+v_{z}\frac{\partial \rho H_{\mathrm{ens}}}{\partial z}=\frac{\partial }{\partial x}\left(k\frac{\partial T}{\partial x}\right)+\frac{\partial }{\partial y}\left(k\frac{\partial T}{\partial y}\right)\\ +\frac{\partial }{\partial z}\left(k\frac{\partial T}{\partial z}\right)+S_{V} \end{array}$$

where *H_ens_* is the mixed enthalpy, *u*_0_ is the velocity component in x direction, *k* is the thermal conductivity, *T* is the temperature, and *S_V_* is the internal heat source term.

### 3.3. Heat Source Model

Given the high energy density of the laser heat source, the resulting weld exhibits a large depth-to-width ratio. In contrast, although the MIG arc heat source has a moderate energy density, it produces a wider heat-affected zone. Since both heat sources are simultaneously active during the process, their effects must be superimposed. For laser–arc hybrid welding, a combined heat source model is adopted, in which the laser energy is represented by a Gaussian rotating cone heat source, and the arc energy is described using a double-ellipsoidal heat source distribution [28,29].

(1) Arc heat source model.

The double-ellipsoid heat source model offers superior capability to replicate the actual molten pool geometry by adjusting parameters such as its shape, heat flow distribution, and heat input [30], with its formula given as follows.

(13) $$q_{af}=\frac{6\sqrt{3}\left(f_{f}\omega_{A}\mathrm{IU}\right)}{a_{f}b_{h}c_{h}\pi \sqrt{\pi }}\exp\left(-\frac{3{\left(x-v_{0}t\right)}^{2}}{a_{f}^{2}}-\frac{3y^{2}}{b_{h}^{2}}-\frac{3z^{2}}{c_{h}^{2}}\right),\;x\geq 0$$

(14) $$q_{\mathrm{ar}}=\frac{6\sqrt{3}\left(f_{r}\omega_{A}\mathrm{IU}\right)}{a_{r}b_{h}c_{h}\pi \sqrt{\pi }}\exp\left(-\frac{3{\left(x-v_{0}t\right)}^{2}}{a_{r}^{2}}-\frac{3y^{2}}{b_{h}^{2}}-\frac{3z^{2}}{c_{h}^{2}}\right),\;x<0$$

where *q_a_*_f_ and *q_ar_* represent the arc power density distribution of the front and rear ellipsoid regions, respectively; *f_f_* and *f*_r_ represent the distribution coefficients of heat before and after the heat source; ω*_A_* denotes the arc thermal efficiency; *I* stands for the welding current; *U* is the arc voltage; *v*_0_ signifies the welding speed; and *a_f_*, *a_r_*, *b_h_*, and *c_h_* are the distribution parameters of the double ellipsoidal heat source, specifically representing the length of the front semi-axis, the length of the rear semi-axis, the half width of the molten pool, and the depth of the molten pool, respectively. For example, in this work, *a_f_* = 2 mm, *a_r_* = 5 mm, *b_h_* = 3.5 mm, and *c_h_* = 3 mm.

(2) Laser heat source model.

Compared with arc heat flow, laser has the characteristics of high energy density and strong penetration. Therefore, compared with the double ellipsoid model, the cone heat source model can better simulate the narrow and deep molten pool morphology formed by laser. The formula is as follows.

(15) $$Q_{\mathrm{laser}}(x,y,z,t)=\frac{9\alpha_{\mathrm{abs}}P}{\pi {R_{0}}^{2}H_{\mathrm{src}}(1-e^{-3})}\exp\left[\frac{-9(x{\left(t\right)}^{2}+y{\left(t\right)}^{2})}{{R_{0}}^{2}\log(H_{\mathrm{src}}/z)}\right]$$

where *Q_laser_*(*x*,*y*,*z*,*t*) is the laser energy density, *P* is the laser power, *H_src_* denotes the height of the heat source, *α_abs_* is the laser absorptivity of the material, and *R*_0_ is the laser beam radius.

### 3.4. Computational Domain and Mesh

In the actual welding process, the assembly error will have a great impact on the flow behavior of liquid metal. In order to clarify the molten pool flow behavior under different assembly conditions, a corresponding mathematical model was established for the welded joints under different assembly errors. For the ideal working condition without assembly error, its core characteristics are not fundamentally different from the gap model, so it is not necessary to model separately.

**Figure 2:**
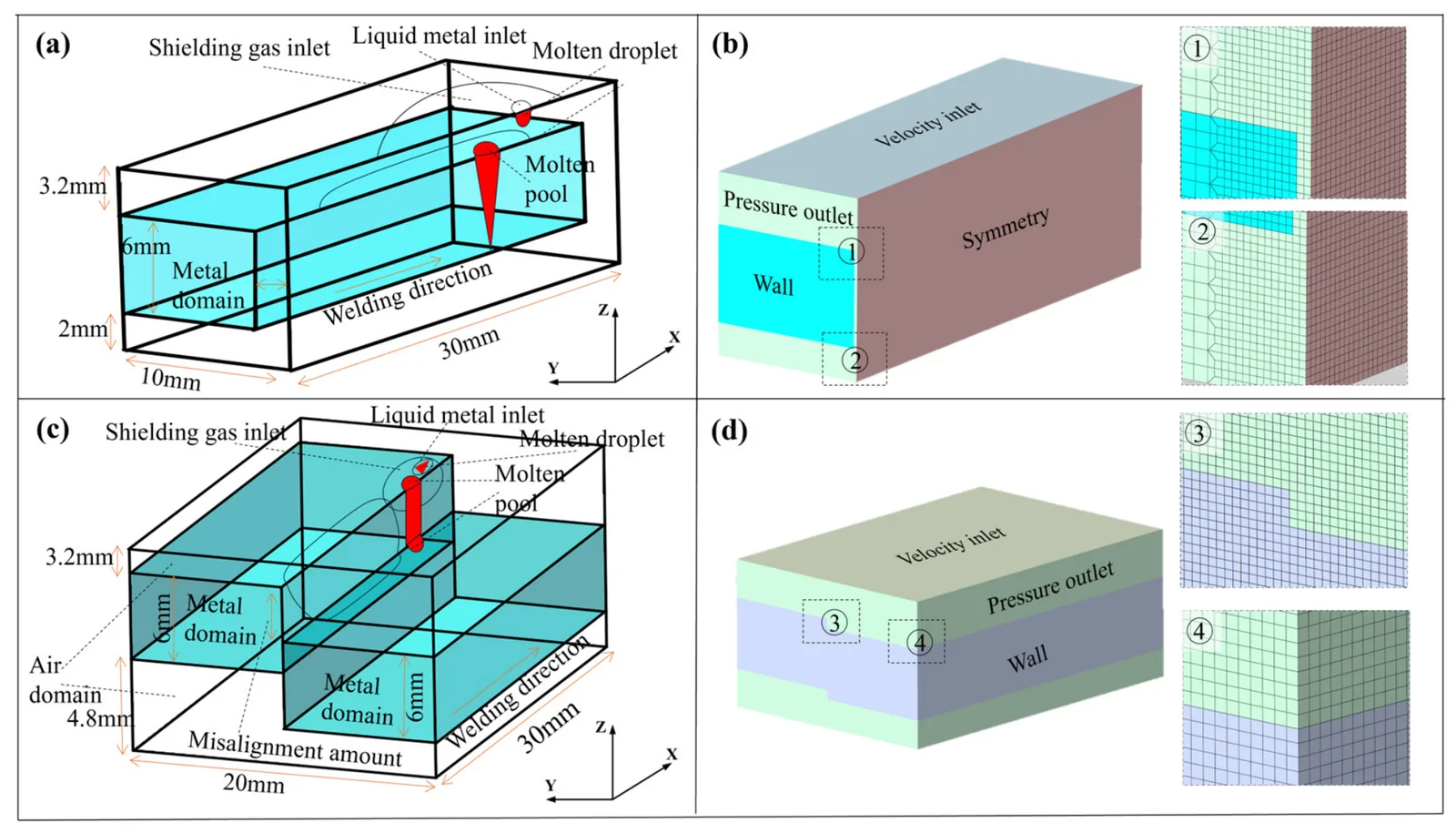
The computational domain and mesh generation of the laser–MIG hybrid welding melt pool model, (**a**) The computational domain of gap conditions. (**b**) The mesh division of gap conditions. (**c**) The computational domain of misalignment conditions. (**d**) The mesh division of misalignment conditions.

In this study, the typical working conditions are selected for numerical simulation so as to avoid the excessive amount of calculation caused by the 150 mm weld. Because the gap molten pool model is symmetrical about the XOZ plane, it can further simplify the calculation and model only half of the calculation domain, as shown in Figure 2a, and the grid division is shown in Figure 2b. This model is applicable to all gap sizes. For the staggered molten pool model, because the weld shape does not have symmetry with respect to the XOZ plane, it is necessary to carry out a global modeling. The calculation domain and grid division are shown in Figure 2c and Figure 2d, respectively. This model is applicable to all the amount of misalignment. The calculation domain is divided into metal domain and air domain. Based on the full penetration welding process adopted in this study, the air domain of the model is divided into two parts. The upper gas domain contains shielding gas, droplets, and weld reinforcement metal, and the lower gas domain contains weld reinforcement metal. The calculation domain of the gap model is discretized by non-uniform hexahedron grid, and the central area of the weld is meshed. The minimum grid size is 0.1 mm; the maximum grid size is 0.4 mm, and the total number of grids is 593,429. Due to the larger size of the staggered model, the minimum size of the basic grid in the weld area is 0.2 mm, and the total number of initial grids is 579,004. In addition, the adaptive grid technology is used to locally densify the molten pool area in the simulation process, and the total number of grids after densification is about 892,451. The upper surfaces of the two models are set as velocity inlets, and all boundaries are pressure outlets except the wall.

### 3.5. Boundary Conditions

(1) Energy boundary condition.

During heat transfer, convection and radiation occur simultaneously on the free surface of the molten pool, subjected to the combined thermal input from the laser and arc. In contrast, only convection and radiation take place on the other wall surfaces, which receive no thermal input. The energy boundary conditions on the free surface and other walls are formulated following the approach of Zhang and Wu [31].

Free surface of molten pool.

−*k*∂*T*/∂*n* = *q_arc_* + *q_laser_* − *q_loss_* (16)

*q_loss_* = [*h_conv_*(*T* − 300) + *εσ*(*T*^4^ − 300^4^) + *ρV_vap_T*] × *CSF* (17)

Other walls.

−*k*∂*T*/∂*n* = −[*h_conv_*(*T* − 300) + *εσ*(*T*^4^ − 300^4^)] × *CSF* (18)

where *q_arc_* and *q_laser_* are the arc and laser heat inputs, *q_loss_* is the heat loss, *h_conv_* is the heat loss, *ε* is the radiation coefficient, *σ* is the Stefan–Boltzmann constant, *σ* = 5.67 × 10^−8^ *V_vap_* is the evaporation coefficient, and *CSF* is the continuum surface force function.

(2) Momentum boundary condition.

In laser–MIG hybrid welding, the fluid flow in the molten pool is driven by the force generated by the arc and is also strongly disturbed by the keyhole formed by the laser [32]. To accurately capture the effect of keyhole dynamic evolution on molten pool fluid flow, the keyhole is modeled in the simulation as a deformation of the molten pool free surface, resulting from the combined action of vapor recoil pressure *P_v_*, arc pressure *P_a_*, and surface tension *P_s_*.

*P* = *P_v_* + *P_a_* + *P_s_* (19)

(1) The arc pressure *P_a_* is the mechanical force generated by arc plasma flow on the surface of molten pool, which is expressed as [33].

(20) $$P_{a}=\frac{\mu_{m}I^{2}}{8\pi \sigma_{j}}\exp\left(\frac{-r^{2}}{2{\sigma_{j}}^{2}}\right)$$

where *μ_m_* represents the vacuum permeability, *r* represents the radial distance from the arc center, *I* represents the welding current, and *a_j_* represents the arc pressure distribution coefficient, satisfying *a_j_* = 0.0005342*I*^0.2684^.

(2) The recoil pressure *P_v_* is the reactive force generated by the high-speed ejection of metal vapor from the surface of the molten pool, which is the main driving force to form the keyhole, and is calculated by the Clausius–Clapeyron equation [34].

(21) $$P_{v}≅0.54P_{0}\exp\left(H_{\mathrm{evp}}\frac{T-T_{g}}{\mathrm{RT}T_{g}}\right)$$

where *H_evp_* is the latent heat of evaporation, *T_g_* is the boiling temperature, *P*_0_ is standard atmospheric pressure, and *R* is the universal gas constant.

(3) The surface tension of the molten pool *P_s_* refers to a intermolecular force acting on the free surface of the molten metal, which is the main force maintaining the shape of the molten pool, and its expression is as follows [35].

*P_S_* = *k_c_γ* (22)

where *γ* is the surface tension coefficient and *k_c_* is the surface curvature determined by the surface normal vector $\vec{n}$. Due to the uneven temperature distribution on the surface of liquid metal, a surface tension gradient, also referred to as Marangoni force, is generated. The expression is as follows [36].

(23) $$F_{Ma}=-\mu \frac{\partial \left(\vec{v}\cdot \vec{s}\right)}{\partial \vec{n}}=\frac{\partial \gamma }{\partial T}\frac{\partial T}{\partial \vec{s}}$$

where $\vec{v}$ and $\vec{s}$ represent the velocity vector and tangent vector on the surface of the molten pool, respectively.

(3) Droplet Transfer.

**Figure 3:**
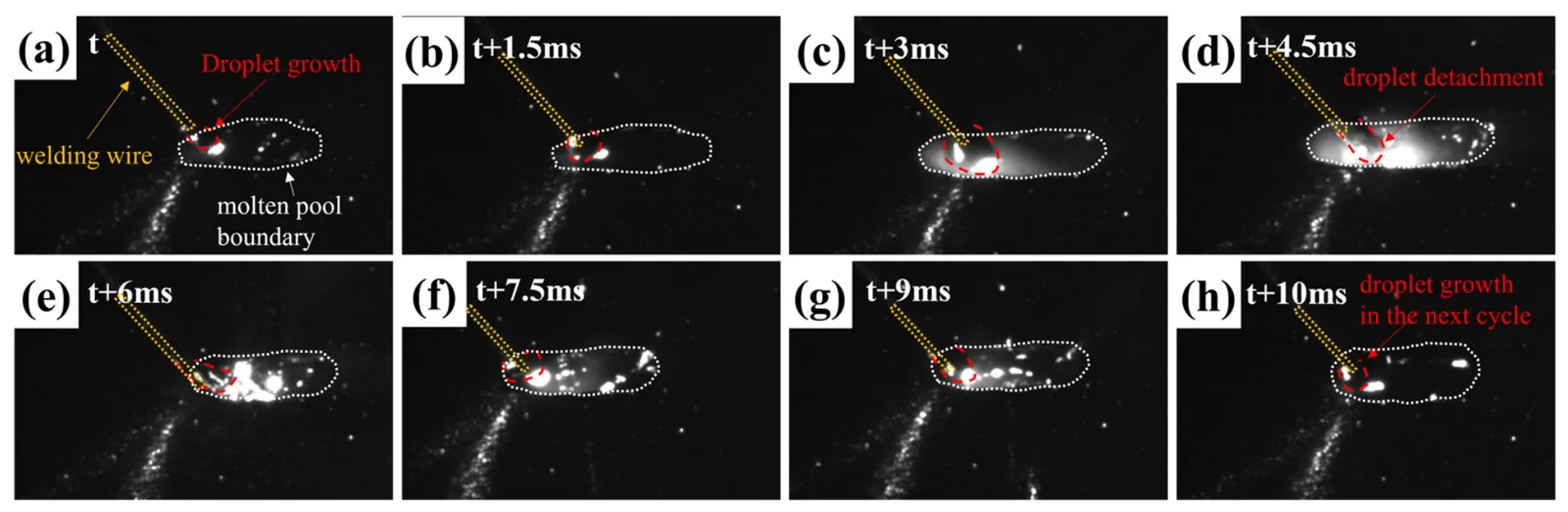
High-speed camera captures droplet transfer at different moments: (**a**) *t* = 0 ms; (**b**) *t* = 1.5 ms; (**c**) *t* = 3 ms; (**d**) *t* = 4.5 ms; (**e**) *t* = 6 ms; (**f**) *t* = 7.5 ms; (**g**) *t* = 9 ms; (**h**) *t* = 10 ms.

In laser–MIG hybrid welding, the MIG torch forms a 60° angle with the laser beam, leading to distinct droplet transfer frequencies and modes compared with conventional arc welding. The welding wire melts to form droplets, which impinge on the molten pool free surface at a specific velocity. This not only modulates molten pool heat flux distribution but also alters its internal fluid flow and keyhole morphology. In this model, droplets are treated as liquid metal with specific mass, temperature, and velocity, entering the molten pool from the welding wire tip at the wire feed speed with periodicity matching the actual droplet transfer period. Specifically, the droplet inflow velocity equals the wire feed speed, and the inflow period corresponds to the actual transfer period. As shown in Figure 3, the droplet transfer period is determined as 10 ms via statistical analysis of the transfer process using high-speed photography.

### 3.6. Tracking of Gas–Liquid Free Surface

The volume of fluid (VOF) approach is used to track the gas–liquid free interface. As shown in Equation (24), for the fluid volume fraction *F* = 1, it denotes a fully liquid grid; 0 < *F* < 1 denotes a gas–liquid interface grid, and *F* = 0 denotes a fully air-filled grid [37].

(24) $$\frac{\partial F}{\partial t}+v_{x}\frac{\partial F}{\partial x}+v_{y}\frac{\partial F}{\partial y}+v_{z}\frac{\partial F}{\partial z}=0$$

### 3.7. Thermophysical Properties of Materials

Table 2 lists the thermophysical parameters of 10CrNiCu steel used in the simulation [38], where the specific heat and thermal conductivity are dependent on the temperature, and others such as density, latent heat of fusion, and coefficient of thermal expansion are constant.

The thermal conductivity property parameters of 10CrNiCu steel vary with temperature as follows.

(25) $$\begin{array}{l} k\left(T\right)=\left\{\begin{array}{l} 39.59\\ 39.59+\frac{41.29-39.59}{160\times (T-300)}\\ 41.29+\frac{27.3-41.29}{630\times (T-460)}\\ 27.3+\frac{35.0-27.3}{610\times (T-1090)}\\ 35 \end{array}\right. \end{array}\;\left({\mathrm{Wm}}^{-1}K^{-1}\right)\;\begin{array}{l} T\leq 300\;K\\ 300\;K<T\leq 460\;K\\ 460\;K<T\leq 1090\;K\\ 1090\;K<T\leq 1700\;K\\ T\geq 1700\;K \end{array}$$

### 3.8. Model Validation

**Figure 4:**
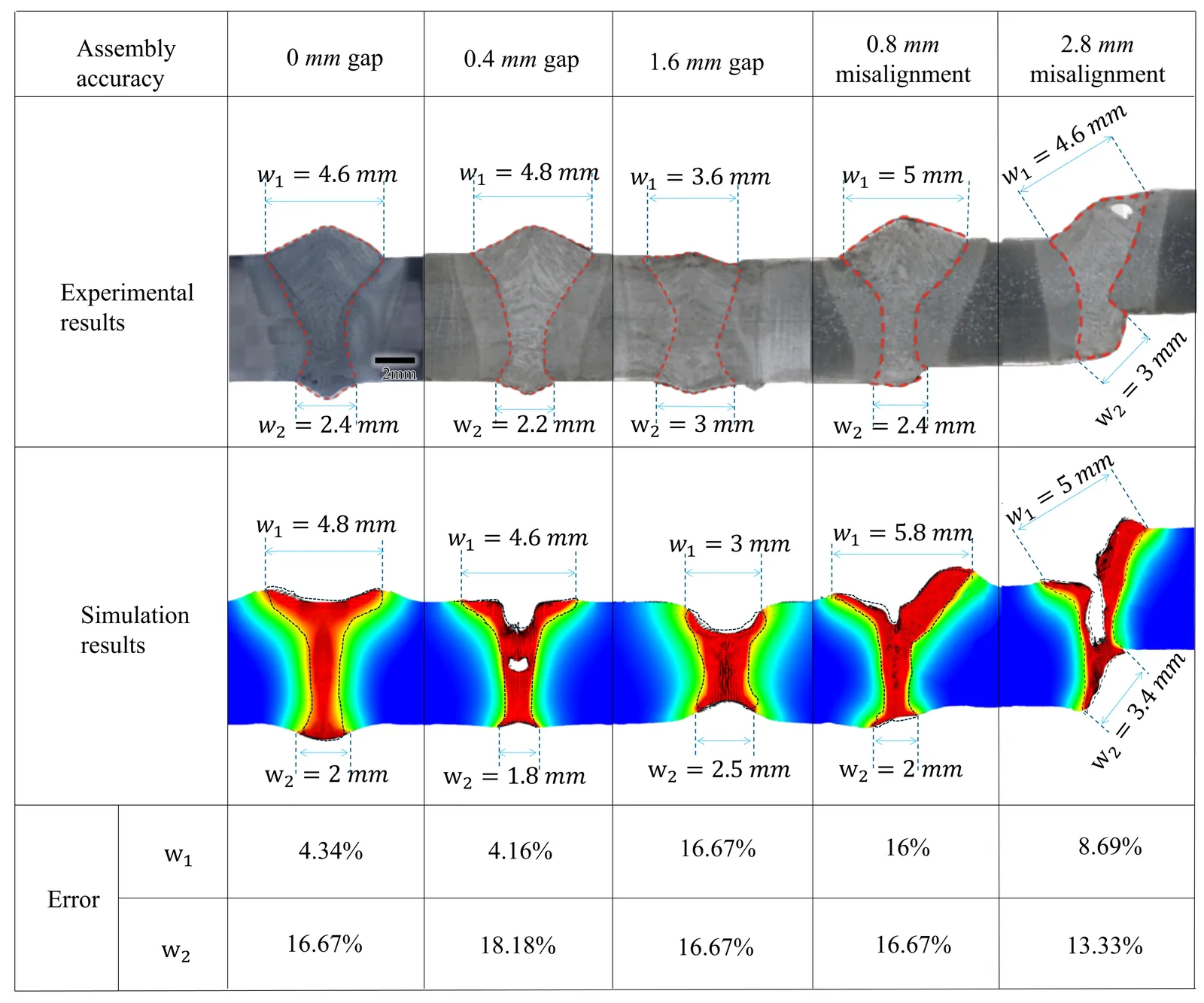
Comparison of simulated cross-sections and molten pool profiles of laser–MIG hybrid welded joints under different assembly conditions.

Based on the welding parameters of 230 a welding current, 4 kW laser power, and 20 mm/s welding speed, the accuracy of the model was verified by comparing the simulation results and experimental results of the molten pool morphology of 10CrNiCu steel under different assembly conditions, the results are shown in Figure 4. A quantitative comparison of simulated molten pool contours with experimental fusion lines revealed the following errors in weld width. For the 0 mm gap condition, the experimentally measured upper weld width was 4.6 mm, and the lower weld width was 2.4 mm, while the simulated upper and lower widths are 4.8 mm and 2.0 mm, corresponding to errors of 4.34% and 16.67%, respectively. For the 0.4 mm gap condition, the experimental upper and lower widths were 4.8 mm and 2.2 mm, while the simulated values are 4.6 mm and 1.8 mm, yielding errors of 4.16% and 18.18%. For the 1.6 mm gap condition, the experimental widths were 3.6 mm for the upper width and 3.0 mm for the lower width, and the simulated widths are 3.0 mm and 2.5 mm, with both upper and lower width errors being 16.67%. For the 0.8 mm misalignment condition, the experimental upper width was 5.0 mm, and the lower width was 2.4 mm, while the simulated upper and lower widths are 5.8 mm and 2.0 mm, giving errors of 16% and 16.67%, respectively. For the 2.8 mm misalignment condition, the measured upper width was 4.6 mm, and the lower width was 3.0 mm, whereas the simulation yields 5.0 mm for the upper width and 3.4 mm for the lower width, resulting in errors of 8.69% and 13.33%, respectively. It can be concluded that the simulation results are in good agreement with the experimental data. While minor discrepancies exist, they are primarily attributed to the substitution of the precise thermophysical properties of the 10CrNiCu steel with those of a similar alloy composition as well as to the simplification of empirical models such as force and heat sources.

## 4. Results and Discussion

### 4.1. Effect of Gap Size on Flow Behaviors of Molten Pool Metal

**Figure 5:**
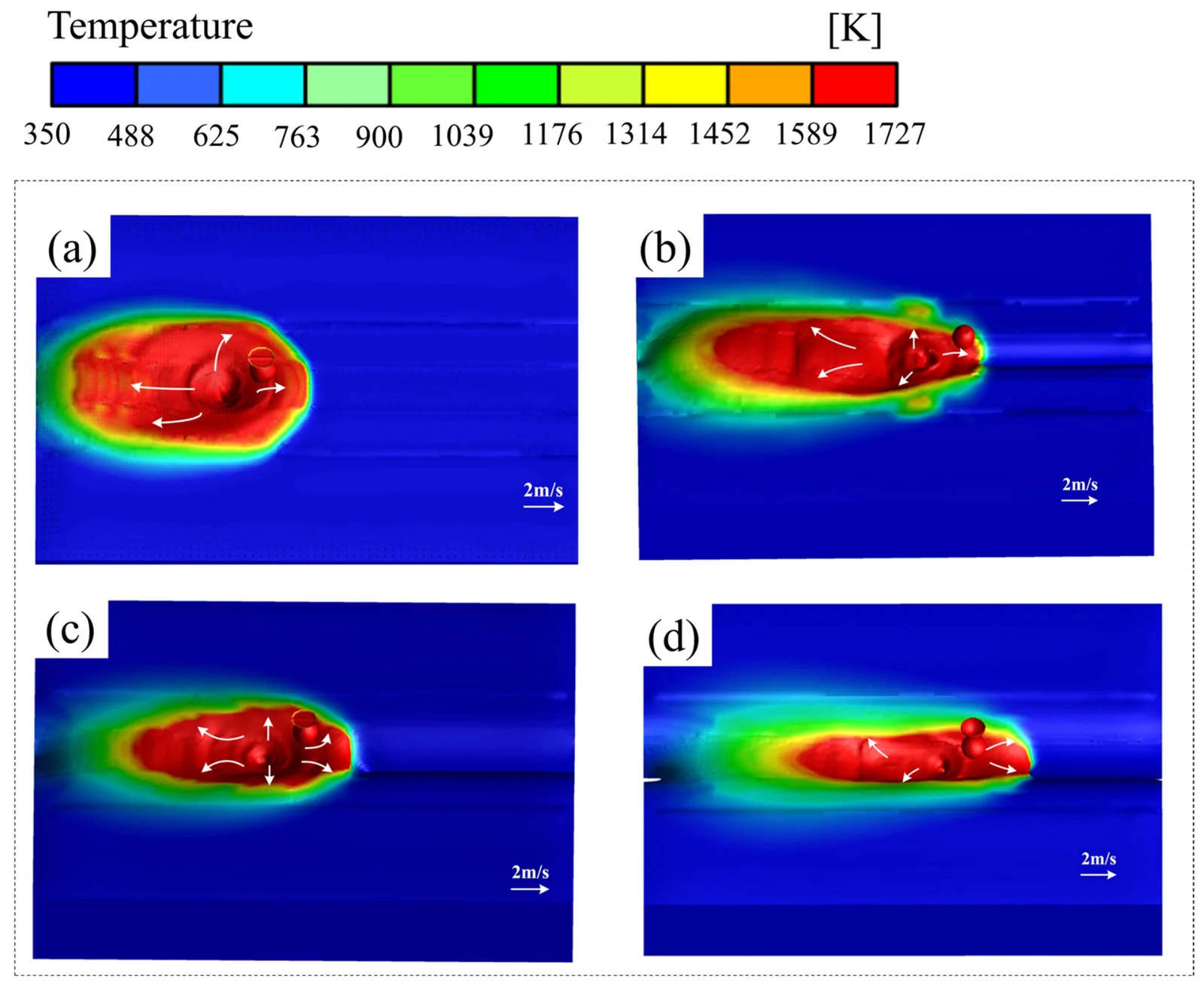
The overall temperature and flow field distributions of the hybrid welded joint molten pool under different gap conditions: (**a**) 0 mm; (**b**) 0.4 mm; (**c**) 0.8 mm; (**d**) 1.6 mm. (White arrows indicate the transient flow direction of the molten metal).

The phenomena described in Section 4.1, Section 4.2, Section 4.3 and Section 4.4 are derived from numerical simulations. To clarify the influence mechanism of gap size on molten pool flow behavior, the molten pool morphology of 10CrNiCu steel under different gaps was investigated. As shown in Figure 5, the overall molten pool flow state exhibits no significant variation with increasing gap size, and the molten pool peak flow velocity remains within 1.5 m/s. At 0 mm gap, as shown in Figure 5a, liquid metal flow within the molten pool stabilizes. Driven by the synergy of molten pool surface tension and dynamic pressure, liquid metal diffuses outward from the keyhole region. Meanwhile, arc pressure disturbs the molten pool front, further facilitating liquid metal spreading and flow. This flow pattern under gap-free conditions is consistent with the classic Marangoni-driven outward flow observed in laser–arc hybrid welding of steel plates, as reported by Zhao et al. [6] for similar material systems. At a 0.4 mm gap, as shown in Figure 5b, molten pool heat flux distribution remains relatively concentrated, and filler metal fully fills the gap to form weld reinforcement. At 0.8 mm gap, as shown in Figure 5c, weld reinforcement decreases. At 1.6 mm gap, as shown in Figure 5d, the molten pool exhibits distinct collapse behavior. The results demonstrate that larger gaps exacerbate molten metal collapse, thereby giving rise to pronounced alterations in the overall molten pool morphology. This gap-induced collapse is physically attributed to the increased free surface area and reduced lateral support from the solid base metal, which weakens the surface tension’s ability to sustain the molten metal weight. Similar bridging limitations with increasing gap size have been documented by Li et al. [12] in CMT welding of aluminum alloys, where the required filler metal volume increases nonlinearly with gap width.

**Figure 6:**
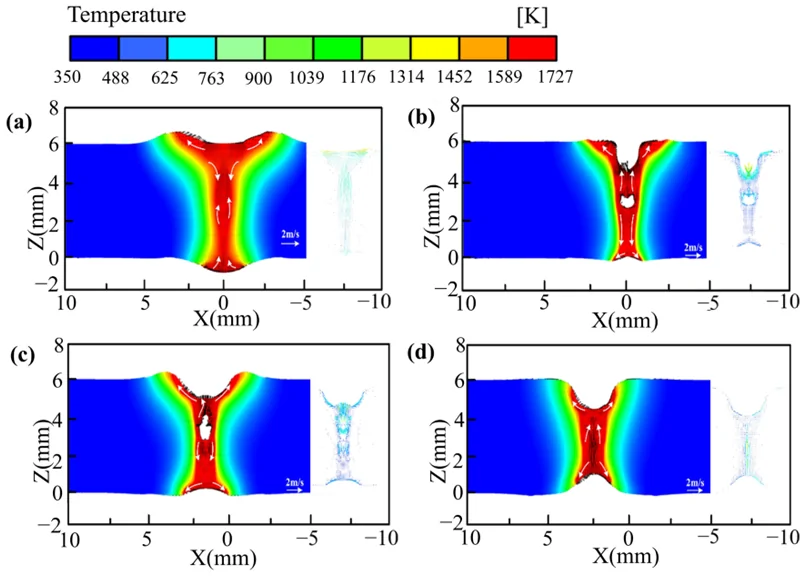
The cross-sectional temperature and flow field distributions of the molten pool under different gap conditions: (**a**) 0 mm; (**b**) 0.4 mm; (**c**) 0.8 mm; (**d**) 1.6 mm. (White arrows indicate the transient flow direction of the molten metal).

Assembly gap is a key factor influencing weld joint quality. At 0 mm gap, as shown in Figure 6a, weld seams solidify gradually during welding with increasing back weld reinforcement, and the overall molten pool flow velocity remains low, with the maximum velocity dropping to approximately 0.22 m/s. At gap sizes from 0.4 to 0.8 mm, as shown in Figure 6b,c, molten pool cross-sections retain the typical “goblet” shape, with only a slight reduction in keyhole depth. The persistence of this goblet morphology at moderate gaps indicates that the vapor recoil pressure remains sufficiently strong to maintain keyhole stability despite the increased flow space. As shown in Figure 6d, at a 1.6 mm gap, keyhole depth decreases significantly; molten pool width widens correspondingly, and molten pool boundaries become nearly linear. This transition from a goblet to a linear boundary reflects a fundamental change in the dominant heat transfer mechanism. At large gaps, the effective thermal contact between the molten pool and the solid base metal is substantially reduced, so heat dissipates more readily through the open gap rather than through downward conduction, which in turn suppresses the deep penetration typically associated with laser keyhole welding. The same trend was noted by Tang et al. [18] in their CFD study of pulsed laser welding with an air gap, where the gap reduced laser energy absorption efficiency. In addition, an increase in the assembly gap results in insufficient liquid metal filling, which inhibits lateral Marangoni flow, weakens the lateral backflow capacity of molten pool liquid metal, and ultimately induces undercut defects.

**Figure 7:**
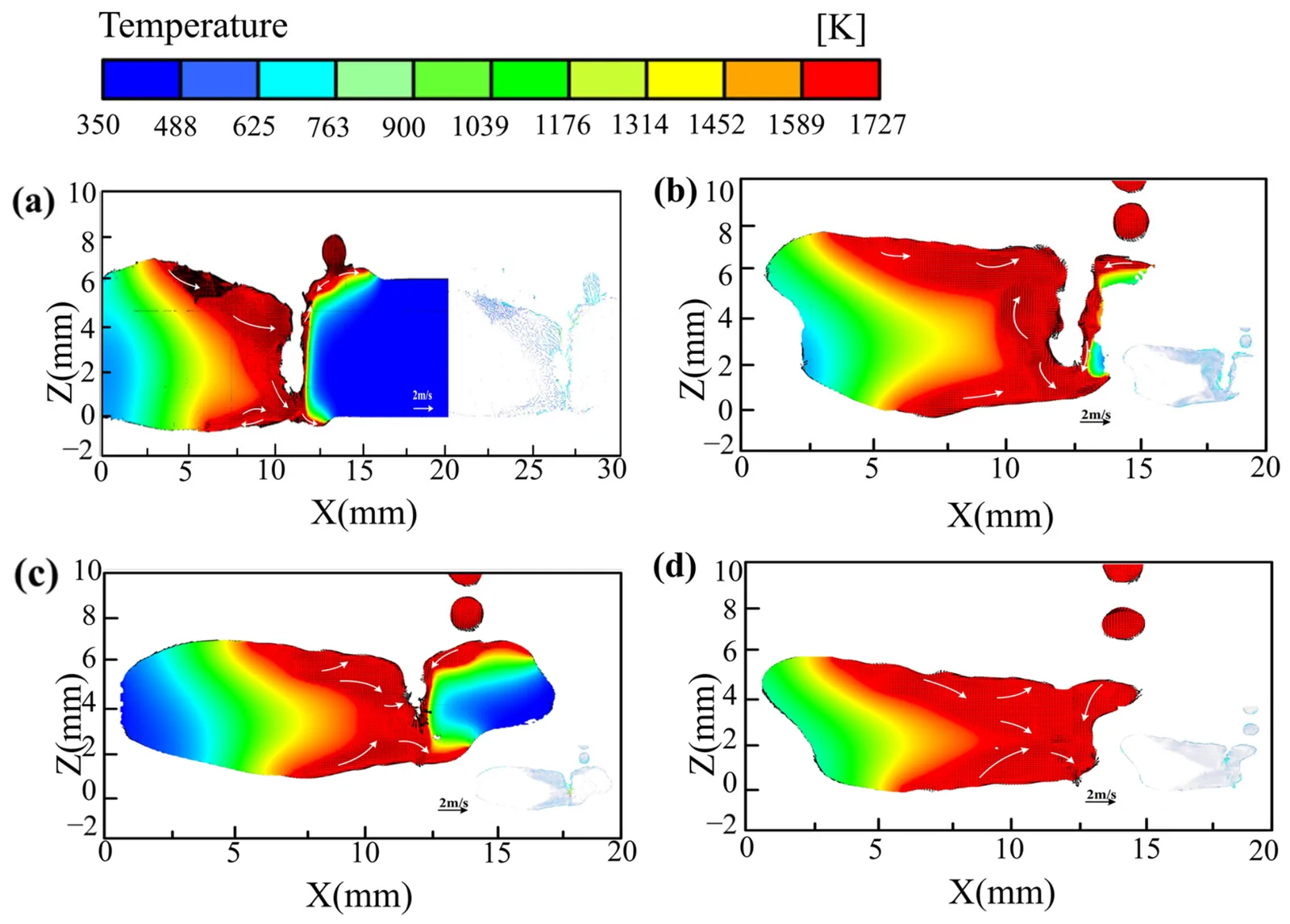
The longitudinal temperature and flow field distributions of the molten pool under different gap conditions: (**a**) 0 mm; (**b**) 0.4 mm; (**c**) 0.8 mm; (**d**) 1.6 mm. (White arrows indicate the transient flow direction of the molten metal).

Molten pool flow behavior exerts a notable regulatory effect on weld formation. When the gap is 0 mm, as shown in Figure 7a, the keyhole penetrates to form a through-hole under the continuous accumulation of vapor recoil pressure, with bubbles in the molten pool, escaping outward immediately. The keyhole bottom then completes its closure. Meanwhile, the liquid metal within the pool keeps recirculating under the combined action of surface tension and hydrodynamic pressure. When the gap is 0.4 mm, as shown in Figure 7b, liquid metal at the molten pool back exhibits a backflow trend and migrates forward, and liquid metal around the keyhole displays bidirectional flow behavior, with some flowing upward driven by vapor recoil pressure and others downward under gravity. This bidirectional flow arises from the competition between the upward vapor recoil pressure and the downward gravitational force, with the relative dominance of each varying spatially around the keyhole. Meanwhile, liquid metal at the molten pool front tends to flow backward. Under this condition, heat distribution at the molten pool front is relatively concentrated, with only a small amount of liquid metal flowing downward and solidifying. The molten pool maintains an overall shape nearly identical to that of the gap-free condition, with a peak flow velocity of 0.9 m/s. When the gap increases to 0.8 mm, as shown in Figure 7c, the arc action zone drives a large number of molten droplets and liquid metal to flow toward the molten pool’s front and lower regions, with rapid solidification occurring and the peak flow velocity dropping to 0.28 m/s. The sharp reduction in flow velocity at this gap size indicates that the increased gap width introduces additional flow resistance, primarily through the disruption of the continuous Marangoni circulation path and the partial loss of liquid metal confinement. When the gap further increases to 1.6 mm, as shown in Figure 7d, molten pool flow behavior becomes gentle, with the peak flow velocity at 0.28 m/s. Since effective heat conduction between liquid metal at the molten pool front and the base metal is impeded by the wide gap, this region’s liquid metal exhibits no obvious solidification tendency, leading to a sagging weld profile and increased susceptibility to underfill defects. These findings are in agreement with the experimental observations of Hao et al. [15] in TIG welding with reserved gaps, where the liquid bridge formation became insufficient when the gap exceeded a critical threshold.

### 4.2. Molten Pool Dynamics in Laser–MIG Hybrid Welding at a Gap Size of 0.8 mm

**Figure 8:**
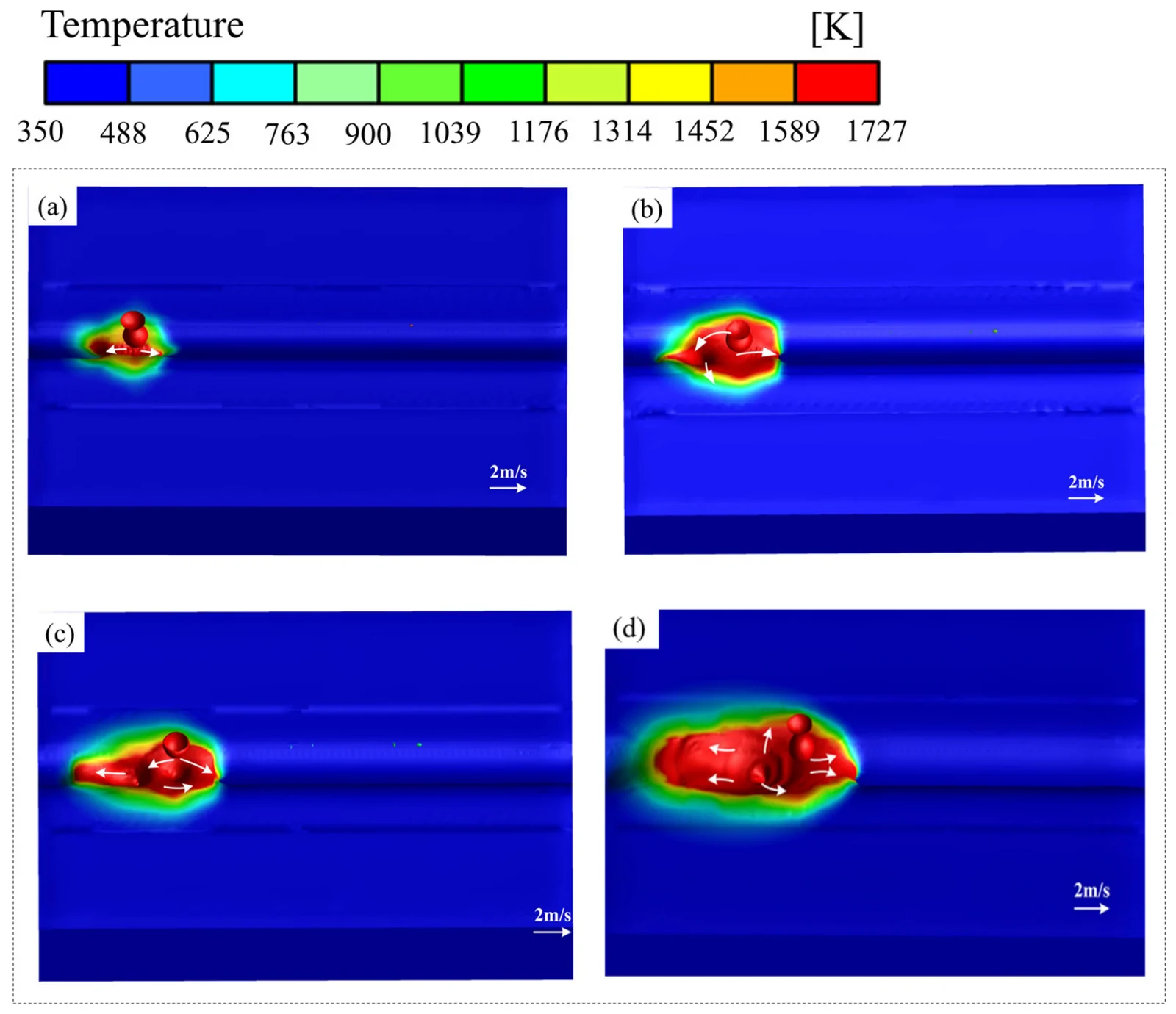
The overall temperature and flow field distributions of the hybrid welded joint molten pool under 0.8 mm gap conditions: (**a**) *t* = 0.042 s; (**b**) *t* = 0.081 s; (**c**) *t* = 0.137 s; (**d**) *t* = 0.291 s. (White arrows indicate the transient flow direction of the molten metal).

To clarify the influence mechanism of assembly gap on molten pool morphology, the dynamic evolution of heat transfer and flow behavior in the 10CrNiCu steel welding process with a 0.8 mm gap is further analyzed, as shown in Figure 8. At t = 0.042 s, as shown in Figure 8a, droplet filling drives molten pool expansion, and the gap provides additional flow space for liquid metal, facilitating its migration toward the pool’s front, rear, and lower regions under the synergistic drive of gravity and surface tension. This accelerated migration is physically due to the reduced constraint from the sidewalls, which lowers the resistance to lateral spreading and allows for gravity to more effectively pull the liquid metal downward into the gap region. At t = 0.081 s, as shown in Figure 8b, liquid metal continues to flow toward the molten pool rear, with a keyhole forming therein. At this stage, liquid metal near the keyhole is more significantly affected by gravity and surface tension, resulting in downward flow from the upper keyhole region. The increased downward flow at the keyhole wall suggests that the gap modifies the local force balance by providing an additional downward pathway for molten metal, which competes with the upward vapor recoil force and may promote keyhole front-wall instability. At t = 0.137 s, as shown in Figure 8c, the molten pool is clearly divided into two distinct regions. The front region is mainly subjected to the combined effects of arc heat and molten droplets, exhibiting a relatively large width. The rear region is dominated by laser heat, superimposed with heat conduction from liquid metal flowing backward from the front pool, leading to a relatively narrow width. This bifurcation of the molten pool into distinct arc-dominated and laser-dominated zones is a typical feature of hybrid welding, but the presence of the gap amplifies the width difference because the gap hinders heat transfer across the joint centerline, thereby reducing thermal coupling between the two zones. At t = 0.291 s, as shown in Figure 8d, liquid metal flow in the molten pool stabilizes. Under the synergistic action of surface tension, molten pool dynamic pressure, and arc pressure, liquid metal spreads and flows uniformly outward from the keyhole region.

**Figure 9:**
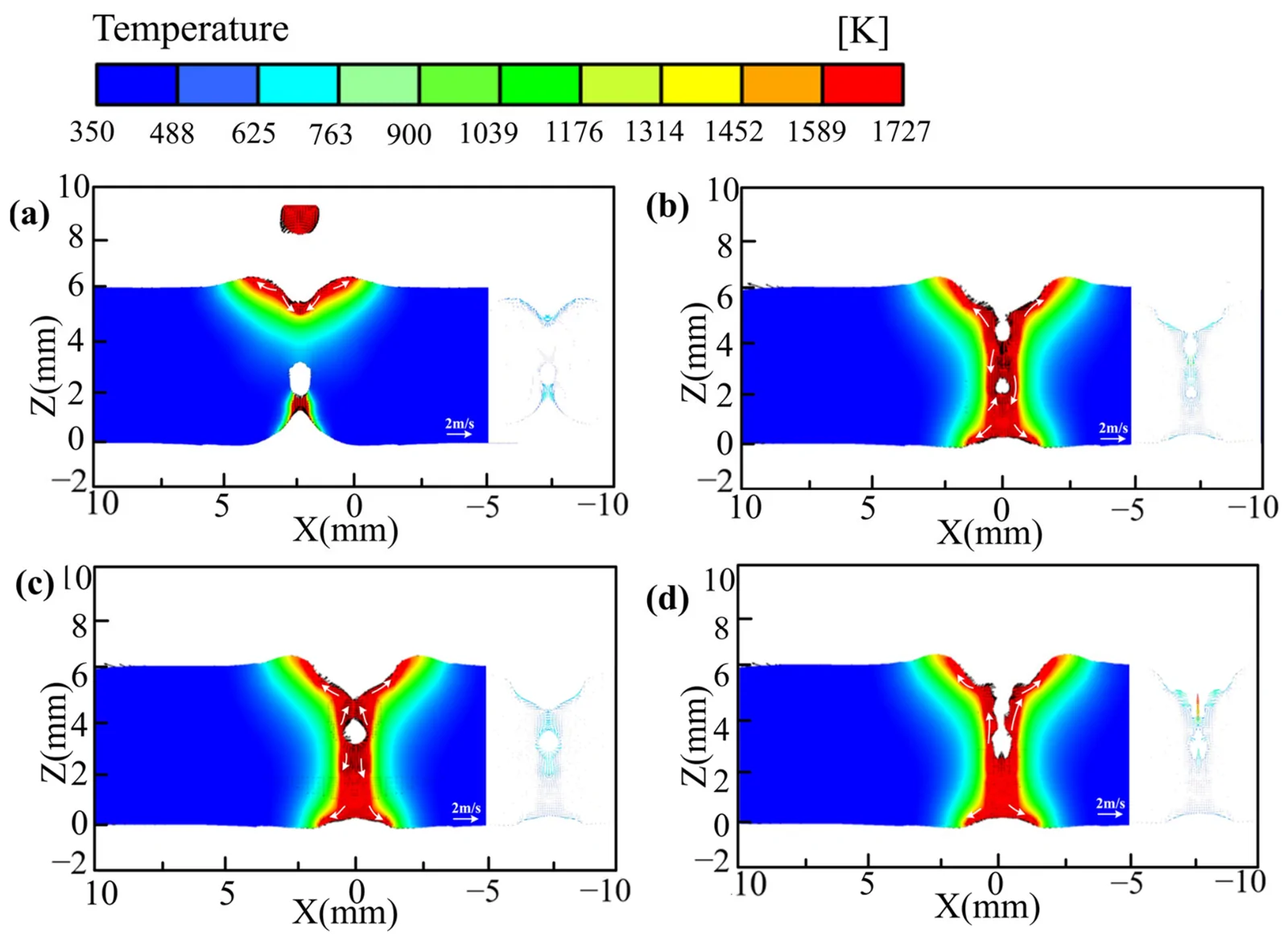
The cross-sectional temperature and flow field distributions of the molten pool under 0.8 mm gap condition (X = 10 mm): (**a**) *t* = 0.257 s; (**b**) *t* = 0.3442 s; (**c**) *t* = 0.3446 s; (**d**) *t* = 0.3454 s. (White arrows indicate the transient flow direction of the molten metal).

The molten pool exhibits differentiated flow characteristics at different times. At t = 0.257 s, as shown in Figure 9a, liquid metal migrates along the welding direction under the synergistic effect of surface tension and molten pool dynamic pressure, forming liquid metal aggregation on the workpiece’s lower surface. In contrast to the molten pool’s central region, the air–liquid contact interface at the pool bottom is larger with a weaker blocking effect. Meanwhile, the air–liquid temperature difference induces enhanced Marangoni convection at the pool bottom, markedly boosting the forward transport capacity of bottom liquid metal. At this time, molten pool peak flow velocity reaches 0.41 m/s. This enhanced bottom convection is physically attributed to the higher temperature gradient at the lower surface, which produces a stronger surface tension gradient and thus more vigorous Marangoni-driven flow. Similar behavior was reported by Zhou et al. [20] in magnetic field-assisted laser welding, where enhanced Marangoni flow contributed to improved gap bridging. At t = 0.3442 s, as shown in Figure 9b, upper keyhole liquid metal is driven upward by vapor recoil pressure, while lower liquid metal spreads downward under gravity. Furthermore, gas entrained during the keyhole’s instantaneous collapse forms discrete bubbles within the molten pool, which rise upward under buoyancy. At t = 0.3446 s, as shown in Figure 9c, bubbles continue to rise under buoyancy dominance. At t = 0.3454 s, as shown in Figure 9d, the rising bubbles merge with the keyhole bottom and ultimately escape the molten pool. The successful bubble escape at this gap size suggests that although the 0.8 mm gap introduces additional flow complexity, the upward buoyancy and vapor recoil pressure remain sufficient to expel the entrained gas before solidification completes, thereby preventing porosity formation. This observation is consistent with the findings of Wu et al. [16] in plasma–MIG hybrid welding, where keyhole bottom collapse led to liquid bridge formation but bubbles could still escape under stable flow conditions.

**Figure 10:**
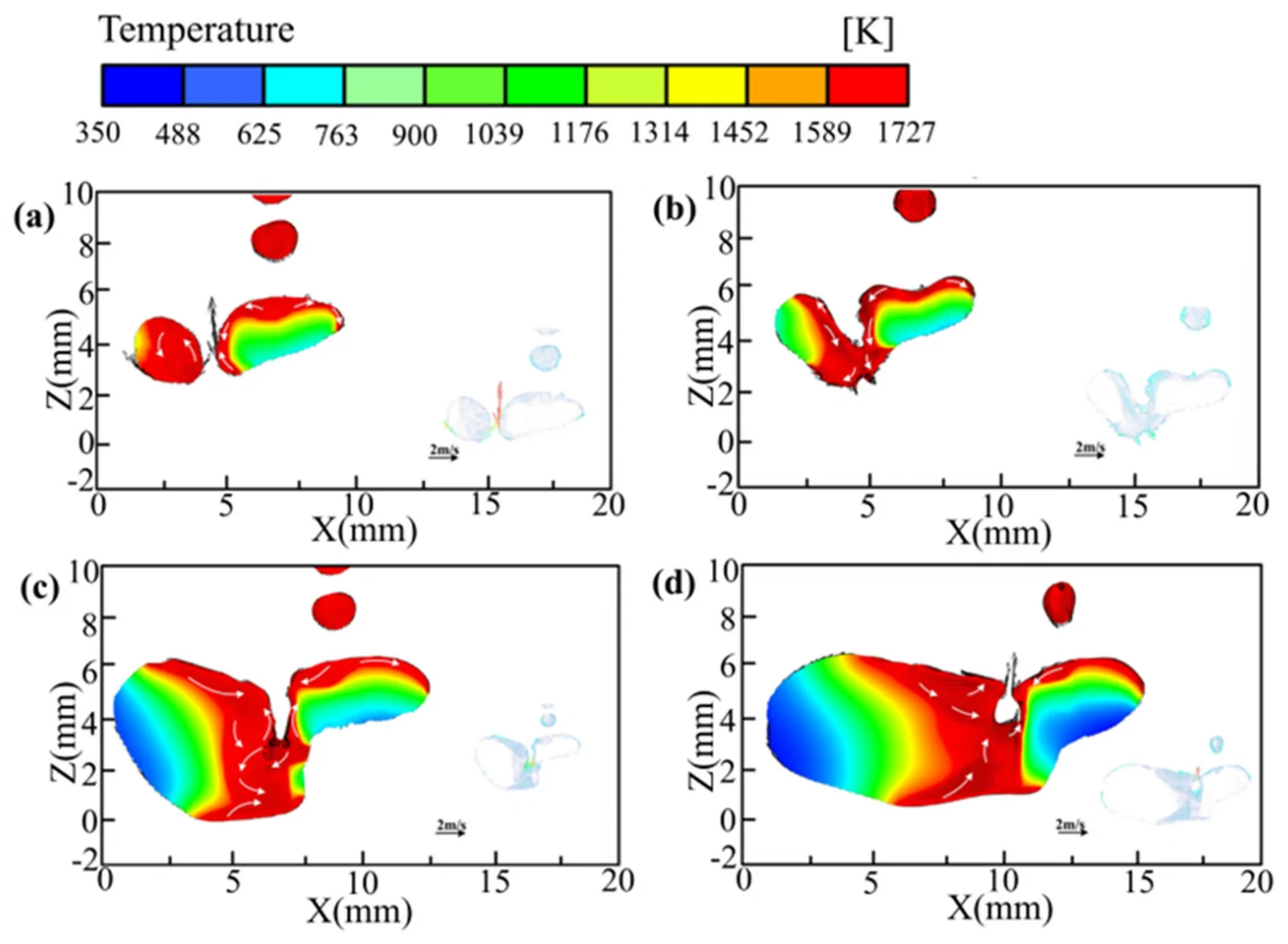
The longitudinal temperature and flow field distributions of the molten pool under 0.8 mm gap conditions: (**a**) *t* = 0.081 s; (**b**) *t* = 0.0978 s; (**c**) *t* = 0.1912 s; (**d**) *t* = 0.3592 s. (White arrows indicate the transient flow direction of the molten metal).

Liquid metal flow behavior differs significantly under multi-force coupling. At t = 0.081 s, as shown in Figure 10a, the keyhole is initially formed, with surrounding liquid metal driven upward by vapor recoil pressure. A counterclockwise vortex forms at the molten pool rear due to gravity simultaneously, and the maximum liquid metal flow velocity in the keyhole region reaches 3 m/s. The formation of this vortex is physically the result of the interaction between the upward vapor-driven flow and the downward gravitational flow. The upward flow along the keyhole front wall is deflected backward at the pool surface, then descends along the rear wall under gravity, completing a recirculation loop. At t = 0.0978 s, as shown in Figure 10b, gravity becomes the dominant factor controlling molten pool flow, and the pool’s liquid metal exhibits an overall downward spreading flow trend. The shift in dominant force from vapor recoil to gravity within a short time interval indicates the highly transient nature of the keyhole stability, which is sensitive to the instantaneous balance of these opposing forces. At t = 0.1912 s, as shown in Figure 10c, the molten pool expands further, and the workpiece is fully molten, with more complex internal flow behavior. Liquid metal on the upper side of the keyhole moves upward, driven by vapor recoil pressure, while that on the lower side flows downward under gravity. Liquid metal at the pool rear migrates toward the keyhole, creating a convection effect with the upward liquid metal flow and eventually forming a counterclockwise vortex behind the keyhole. At t = 0.3592 s, as shown in Figure 10d, the peak flow velocity of the molten pool remains at 2.7 m/s in the keyhole region. Meanwhile, the downward flow of liquid metal is suppressed by surface tension, and the liquid metal at the pool bottom begins to flow back upward. This upward reversal at the pool bottom is a direct consequence of the surface tension force, which acts to minimize the free surface area and counteracts the gravitational driving force when the pool depth becomes sufficiently large relative to the gap width. This dynamic competition between gravity and surface tension has been similarly discussed by Chen et al. [17] in the context of liquid bridge formation in pulse laser welding.

### 4.3. Effect of Misalignment Size on Flow Behaviors of Molten Pool Metal

**Figure 11:**
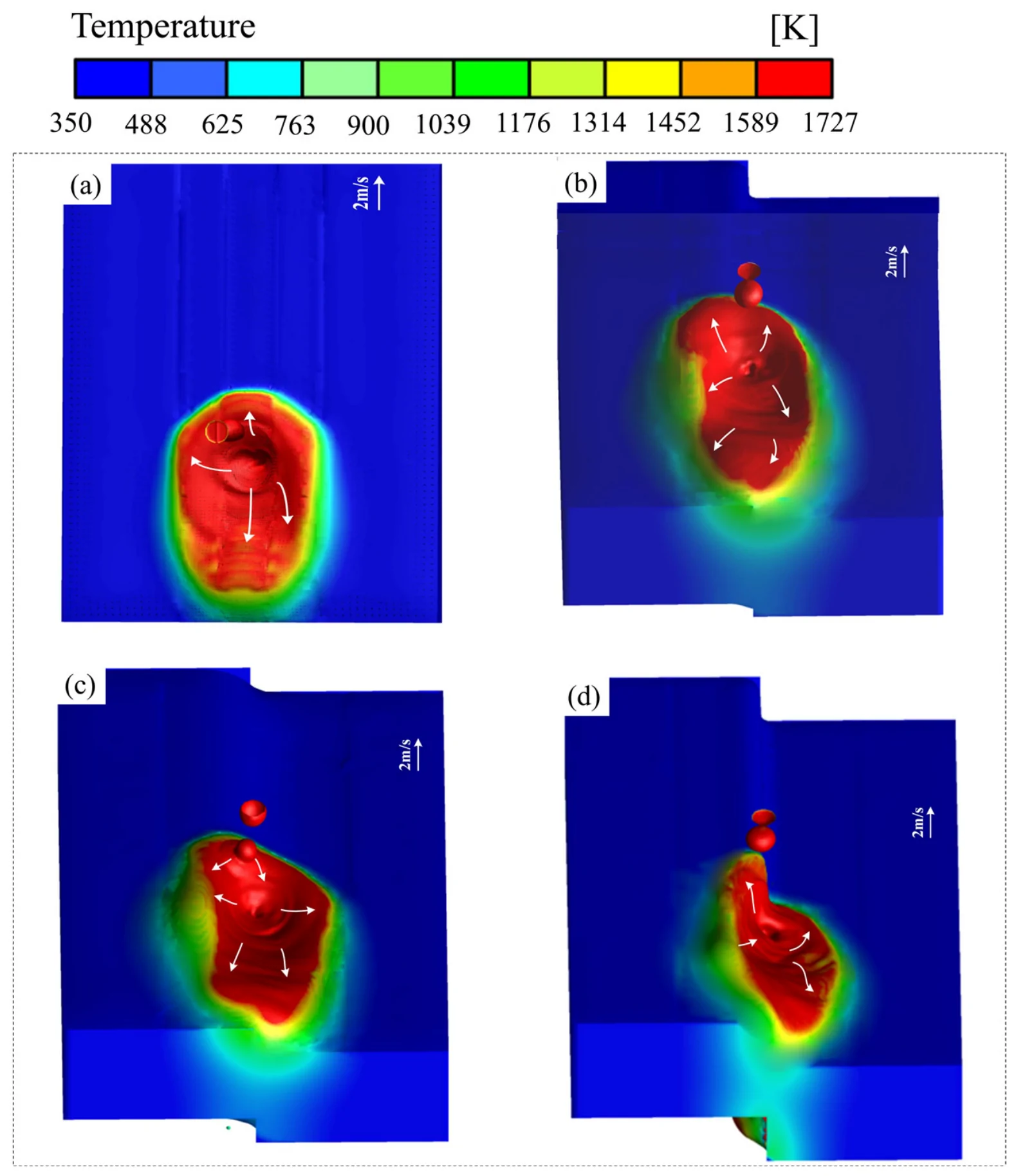
The overall temperature and flow field distributions of the hybrid welded joint molten pool under different misalignment conditions: (**a**) 0 mm; (**b**) 0.8 mm; (**c**) 1.6 mm; (**d**) 2.8 mm. (White arrows indicate the transient flow direction of the molten metal).

To clarify the influence mechanism of misalignment size on molten pool flow behavior, the thermal field distribution and metal flow behavior of 10CrNiCu steel under different misalignment values were investigated, as shown in Figure 11. At misalignment values from 0 to 0.8 mm, as shown in Figure 11a,b, molten pool liquid metal spreads and flows centered on the keyhole, with the overall molten pool morphology exhibiting little difference from the misalignment-free state. The velocity of liquid metal migrating from the low to high side of the misaligned joint is approximately 0.83 m/s at this stage. This relatively symmetrical flow pattern at small misalignment indicates that the step height is insufficient to disrupt the dominant Marangoni and vapor recoil-driven circulation, and the liquid metal can still bridge the height difference without significant directional bias. At a 1.6 mm misalignment, as shown in Figure 11c, the molten pool shows a distinct asymmetric shape. Cumulative heat buildup on the high side forms a large volume of molten metal, which undergoes lateral flow and gradual accumulation under surface tension, ultimately forming weld reinforcement. The asymmetry arises because the high side of the joint intercepts more arc heat and droplet momentum, creating a thermal imbalance across the weld centerline. At a further increased misalignment of 2.8 mm, as shown in Figure 11d, the molten pool width on the high side of the joint decreases significantly. This occurs as gravity drives a large amount of molten metal toward the low side, which partially inhibits the lateral flow of Marangoni flow, reducing the velocity of liquid metal migrating from the low to high side to 0.48 m/s. The reduction in cross-flow velocity with increasing misalignment is a direct consequence of the steepened gravitational potential gradient, which diverts a greater fraction of the molten metal downward rather than laterally, thereby weakening the spreading capacity and promoting asymmetrical solidification patterns.

**Figure 12:**
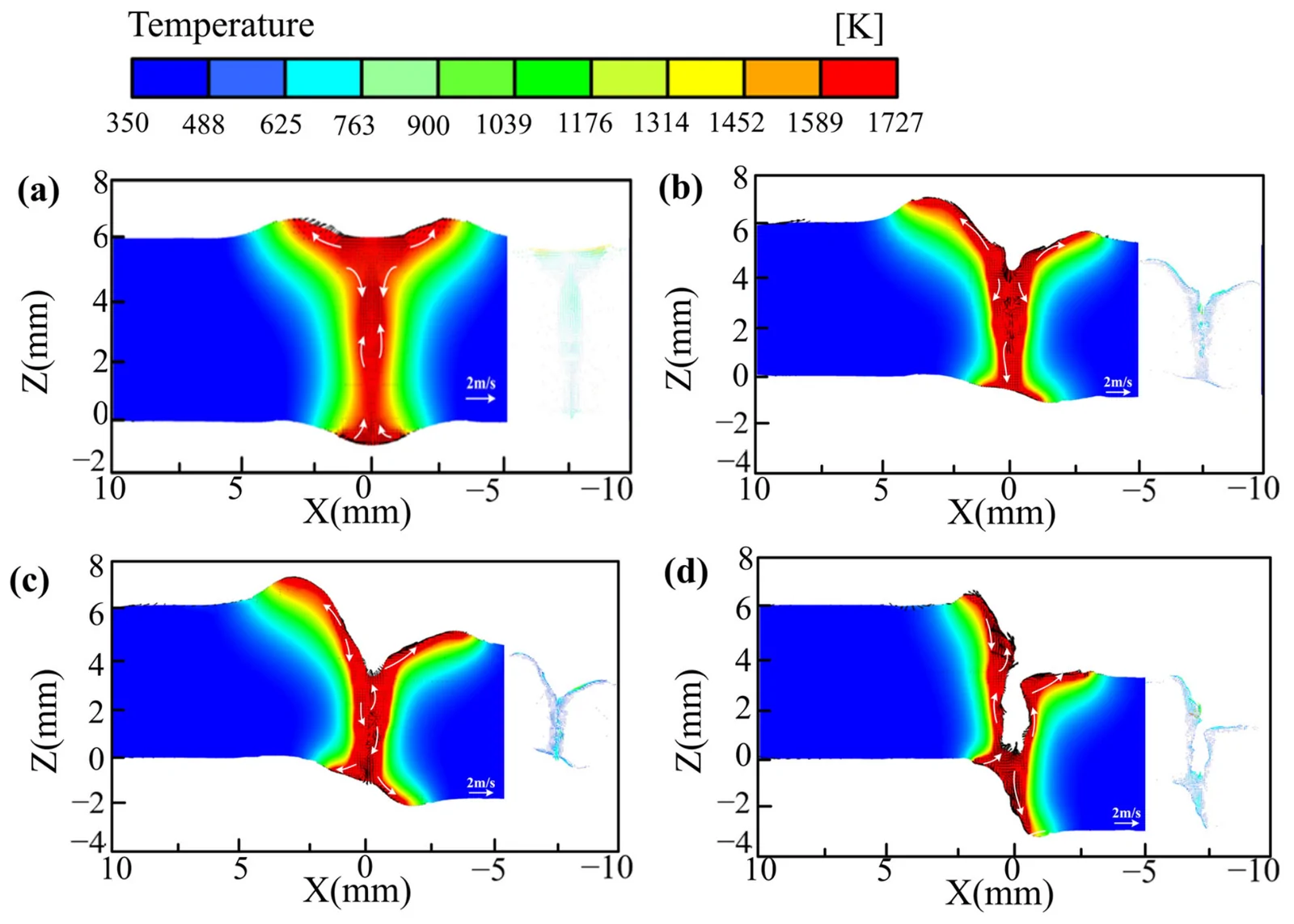
The cross-sectional temperature and flow field distributions of the molten pool under different misalignment conditions: (**a**) 0 mm; (**b**) 0.8 mm; (**c**) 1.6 mm; (**d**) 2.8 mm. (White arrows indicate the transient flow direction of the molten metal).

With increasing misalignment, liquid metal flow behavior and force distribution characteristics within the molten pool change markedly. At 0.8 mm misalignment, as shown in Figure 12b, liquid metal around the keyhole flows upward along the keyhole driven by vapor recoil pressure and diffuses toward the workpiece sides via Marangoni forces. Liquid metal in the molten pool’s lower region flows downward under gravity. At 1.6 mm misalignment, as shown in Figure 12c, liquid metal on the assembly’s high side tends to flow downward under gravity at a velocity of approximately 0.27 m/s. The emergence of this directional downward flow at 1.6 mm misalignment indicates that the gravitational potential difference across the step has become sufficient to overcome the surface tension pinning effect that normally retains liquid metal on the high side. At a further increased misalignment of 2.8 mm, as shown in Figure 12d, molten pool flow behavior grows more complex. Liquid metal on the assembly’s high side flows toward the low side under the synergistic effect of gravity and surface tension, reaching a velocity of up to 0.43 m/s. Upward-flowing liquid metal near the keyhole forms convection with the downward-flowing stream, inducing unstable keyhole wall conditions that favor collapse and pore formation. This convective interaction is physically critical. The downward stream from the high side impinges upon the upward vapor-driven flow along the keyhole wall, creating a shear layer that erodes the keyhole wall and promotes localized collapse. The resulting keyhole instability increases the entrapment of shielding gas and metal vapor, which cannot readily escape due to the asymmetric flow field, thus elevating porosity risk.

**Figure 13:**
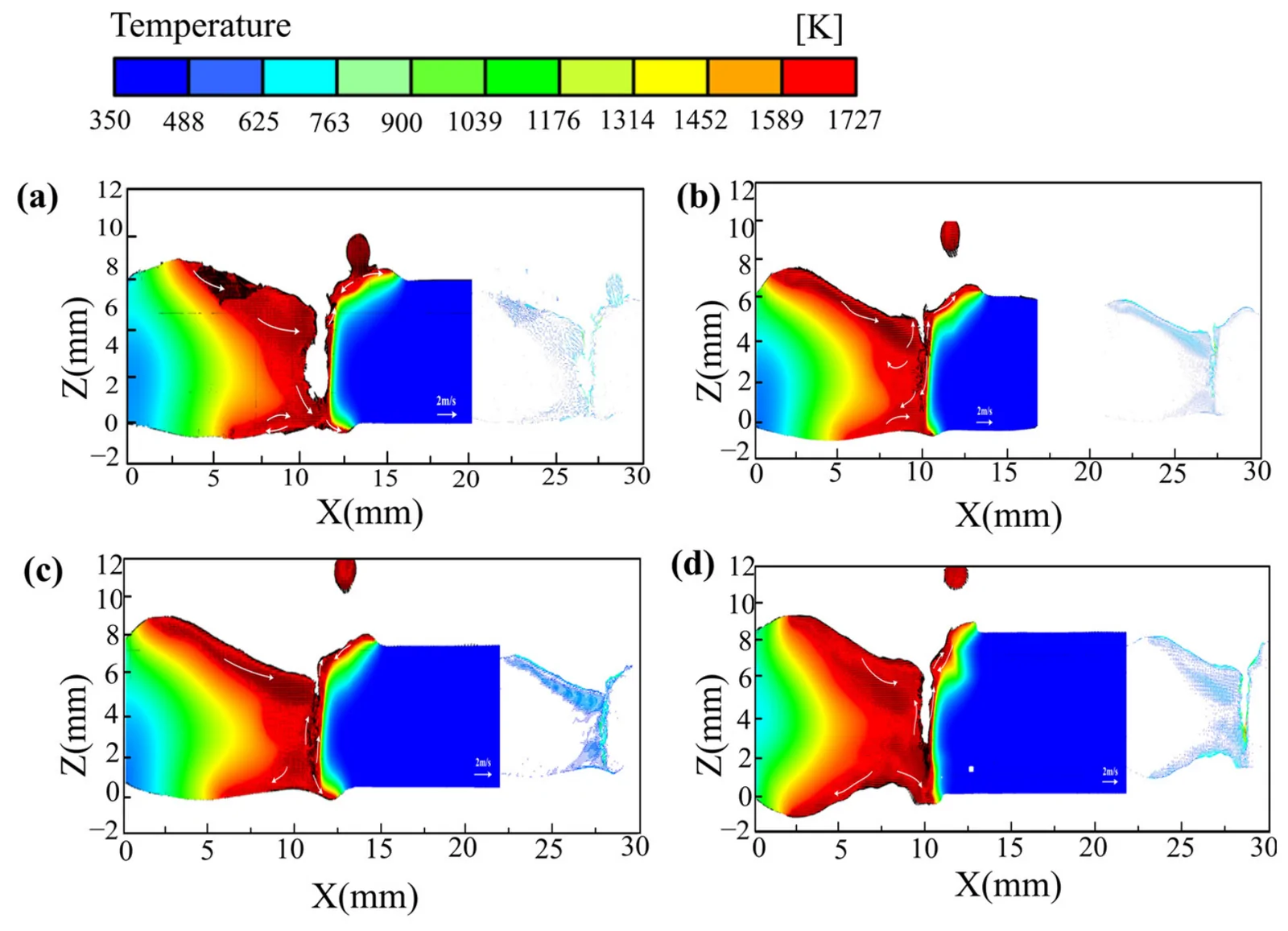
The longitudinal temperature and flow field distributions of the molten pool under different misalignment conditions: (**a**) 0 mm; (**b**) 0.8 mm; (**c**) 1.6 mm; (**d**) 2.8 mm. (White arrows indicate the transient flow direction of the molten metal).

Observing the temperature and flow field distributions on the longitudinal cross-section of the molten pool under different misalignment conditions, as shown in Figure 13, it can be found that at low misalignment values, molten pool flow behavior is highly consistent, with liquid metal reaching a maximum velocity of approximately 1.4 m/s. Specifically, liquid metal around the keyhole surges upward under vapor recoil pressure. That in the upper molten pool flows backward under surface tension traction, while that in the middle and rear regions bends diagonally downward under gravity. That in the lower molten pool spreads bilaterally under the synergistic effect of surface tension and molten pool dynamic pressure. This characteristic flow pattern resembles the classic circulation observed in full-penetration laser welding, where the keyhole acts as a central pump driving both upward and outward flow. When misalignment further increases to 2.8 mm, as shown in Figure 13d, the molten pool expands in overall volume accordingly. Most molten metal in the pool front flows downward under gravity to fill the pool. Surface tension’s regulatory effect on molten pool flow is relatively weakened, thus attenuating the forward flow tendency of liquid metal. The attenuation of surface tension influence at large misalignment can be explained by the increased pool depth and volume, which reduce the surface-to-volume ratio and therefore diminish the relative importance of surface-tension-driven Marangoni flow compared to gravity-driven bulk flow. At the same time, increased misalignment elevates keyhole depth, markedly raising the probability of pore formation induced by keyhole instability and collapse.

### 4.4. Molten Pool Dynamics in Laser–MIG Hybrid Welding at a Misalignment of 1.6 mm

**Figure 14:**
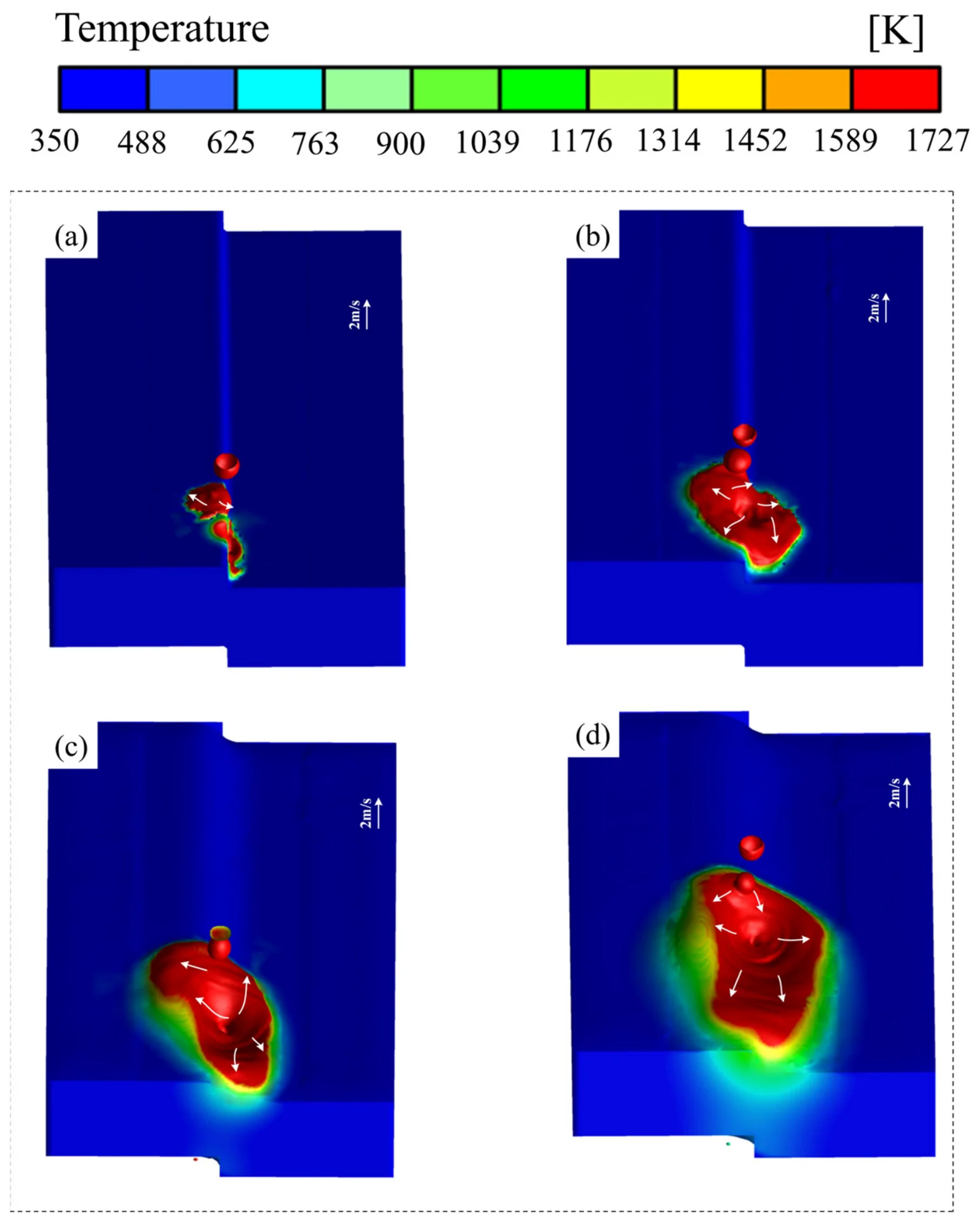
The overall temperature and flow field distributions of the hybrid welded joint molten pool under 1.6 mm misalignment conditions: (**a**) *t* = 0.0168 s; (**b**) *t* = 0.104 s; (**c**) *t* = 0.2 s; (**d**) *t* = 0.466 s. (White arrows indicate the transient flow direction of the molten metal).

Similar to the assembly gap, a misalignment size of 1.6 mm is selected to analyze the dynamic evolution of heat transfer and flow behavior in the molten pool under misalignment error. In the initial stage of welding, as shown in Figure 14a, the laser and arc heat sources have not yet formed an effective coupling relationship, acting independently on the workpiece surface to form separate molten pools. At this stage, in the shallow molten pool formed by the arc heat source, liquid metal is subjected to the combined effect of arc pressure and droplet impact force, with the liquid metal on the assembly’s high side exhibiting a distinct lateral spreading trend. The geometric constraint of the staggered structure induces a vertical downward flow tendency in some liquid metal as it passes through the step region. This early-stage decoupling is typical in hybrid welding before the heat sources establish a stable interaction zone, but the misalignment exacerbates the asymmetry by positioning the two plates at different heights, which alters the effective standoff distance from the arc to each plate and thus changes the local heat input distribution. At t = 0.104 s, as shown in Figure 14b, the separate molten pools formed by the two heat sources start to couple, and the liquid metal flow on the molten pool surface distributes as an outward spread centered on the keyhole. At t = 0.2 s, as shown in Figure 14c, the stepped geometry of the staggered structure at the weld joint exerts a notable regulatory effect on the overall molten pool morphology. Since arc heat energy is concentrated primarily on the assembly’s high side, the heat input on the low side is relatively insufficient, which significantly restricts molten pool expansion and ultimately results in a distinct asymmetric molten pool morphology. The thermal asymmetry is physically unavoidable because the arc plasma preferentially flows toward the closer high-side plate surface, reducing the effective heating of the low side and creating a thermal gradient across the gap. This phenomenon has been similarly observed by Song et al. [8] in hybrid welding of thick steel sections, where uneven heat distribution led to asymmetric weld profiles. At t = 0.466 s, as shown in Figure 14d, the molten pool expands further in coverage, with the internal liquid metal flow state gradually stabilizing.

**Figure 15:**
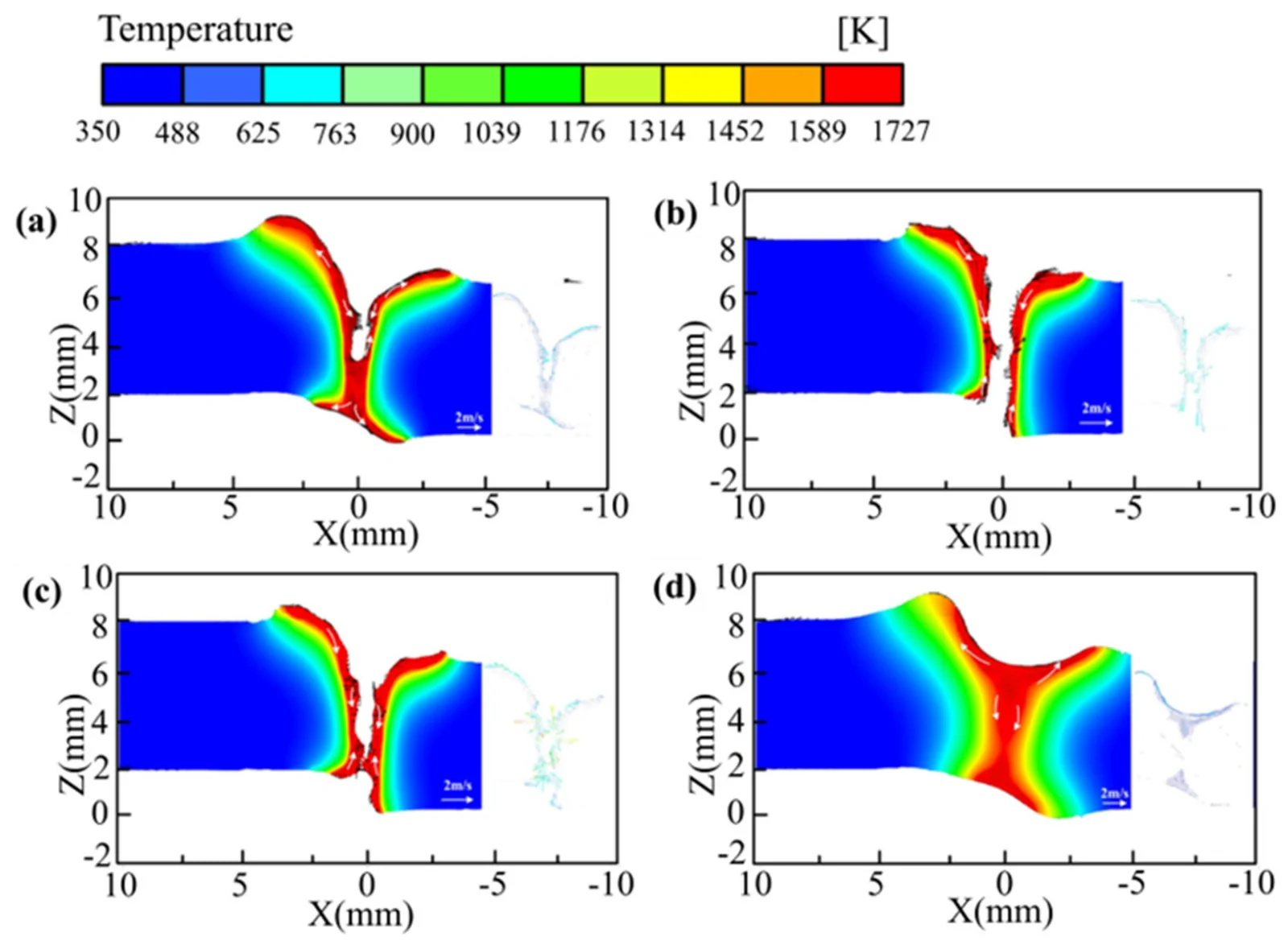
The cross-sectional temperature and flow field distributions of the molten pool under 1.6 mm misalignment condition (X = 10 mm): (**a**) *t* = 0.3412 s; (**b**) *t* = 0.344 s; (**c**) *t* = 0.346 s; (**d**) *t* = 0.469 s. (White arrows indicate the transient flow direction of the molten metal).

Keyhole morphology and molten pool flow behavior evolve dynamically with the welding process. At t = 0.3412 s, as shown in Figure 15a, the laser heat source moves past the monitoring point. Liquid metal around the keyhole is driven upward by vapor recoil pressure, then flows back downward along the molten pool walls. Affected by the joint geometry, the keyhole is relatively deep, and liquid metal flowing down from the assembly’s high side induces severe disturbance to the keyhole. The downward flow velocity of liquid metal at the keyhole opening reaches 0.59 m/s, and the keyhole is thus at risk of collapse. The increased keyhole depth under misalignment is the result of the compounded effects of the elevated step height, which allows for deeper laser penetration due to reduced plasma shielding on the high side, and the altered melt pool geometry, which reduces lateral support around the keyhole. At t = 0.344 s, as shown in Figure 15b, the supporting effect of vapor recoil pressure converts the keyhole to a through-hole state, and liquid metal at the molten pool bottom exhibits synchronous upward reflux. At t = 0.346 s, as shown in Figure 15c, vapor recoil pressure gradually diminishes, failing to maintain the through-hole’s structural stability. Liquid metal at the keyhole bottom contracts inward preferentially under surface tension, forming a locally connected liquid bridge structure that provides an initial “overlap point” for the closure of the keyhole bottom. At t = 0.469 s, as shown in Figure 15d, the laser heat source has moved away from the monitoring point. Liquid metal flow from the high-temperature to low-temperature zone persists in the upper molten pool, while liquid metal in the pool’s middle region flows downward, driven primarily by gravity.

**Figure 16:**
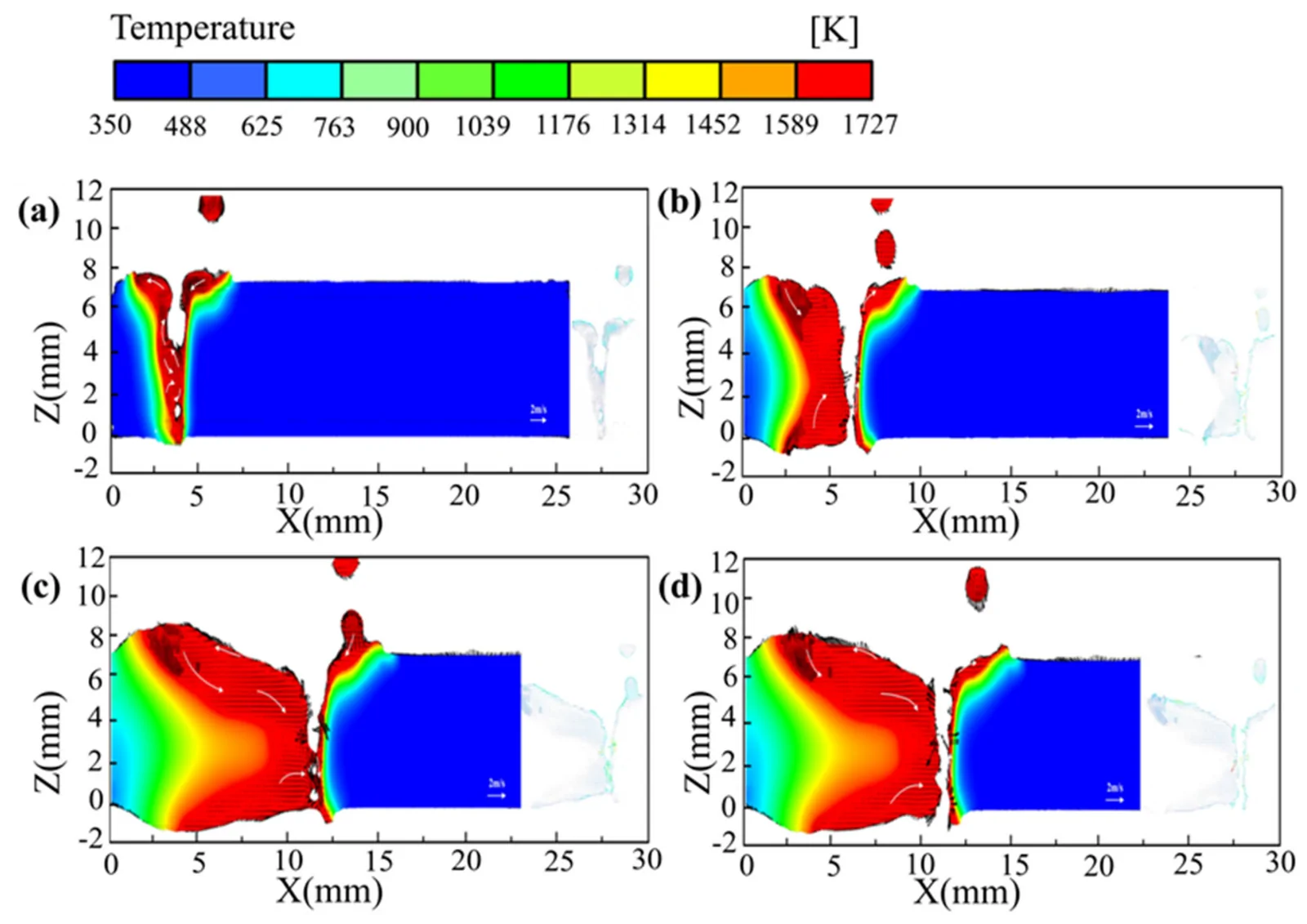
The longitudinal temperature and flow field distributions of the molten pool under 1.6 mm misalignment conditions: (**a**) *t* = 0.0778 s; (**b**) *t* = 0.203 s; (**c**) *t* = 0.475 s; (**d**) *t* = 0.476 s. (White arrows indicate the transient flow direction of the molten metal).

Molten pool flow behavior and keyhole stability undergo complex dynamic changes under misalignment conditions. At t = 0.0778 s, as shown in Figure 16a, the arc heat source forms a shallow molten pool ahead of the keyhole. Liquid metal within the pool migrates backward under the combined effect of arc pressure and droplet impact force, interfering with liquid metal flow inside the keyhole. Instability-induced keyhole wall collapse induces bubble formation in the molten pool, and the pool’s liquid metal flow characteristics change markedly at this stage. Downward-flowing liquid metal collides with upward-flowing liquid metal driven by Marangoni forces, forming two counterrotating vortex structures in the molten pool’s middle region. The formation of these counterrotating vortices is a direct indicator of severe flow disruption. The downward stream from the high side and the upward stream from the keyhole interact to create a stagnation zone where liquid metal accumulates and rotates, which not only delays bubble escape but also intensifies local thermal gradients. As the welding process proceeds, the molten pool expands further in volume, with its flow state largely consistent with that under misalignment-free conditions. The keyhole bottom alternates between open and closed states, as shown in Figure 16b. Due to the special joint geometry, the keyhole is prone to instability with concomitant bubble formation, as shown in Figure 16c. When the keyhole reverts to a through-hole configuration again, the generated bubbles escape the molten pool, as shown in Figure 16d. The results indicate that misalignment error is a significant factor responsible for the disturbed molten pool behavior, unstable keyhole dynamics, and the formation of porosity defects during hybrid welding.

### 4.5. Assembly Error Adaptability of Laser–MIG Hybrid Welding for 10CrNiCu Steel

Before presenting the tolerance results, it is necessary to define the criteria used to determine the acceptable limits for gap and misalignment. In this study, the gap tolerance is defined as the maximum assembly gap at which the weld can maintain a sound surface formation without collapse, undercut, or lack of fusion. Specifically, when the gap exceeds a critical value, the molten metal’s bridging capacity drops sharply, surface tension fails to support its weight, and the molten metal collapses entirely under gravity, leading to weld collapse and the formation of collapse cavities on the weld surface. The gap tolerance is therefore determined by visual inspection of the weld surface morphology; the critical gap at which collapse cavities first appear is recorded as the tolerance limit. Table 3 lists the weld surface morphology and gap tolerance for gap deviations under different welding currents. In the initial welding stage over a certain distance, the joint gap was narrow, and the weld formed well consistently. When the gap reached a critical value, the molten metal’s bridging capacity dropped sharply. The molten pool could not be fully filled; surface tension failed to support its weight, and molten metal collapsed entirely under gravity, triggering weld collapse. This catastrophic failure of the liquid bridge occurs when the gap width exceeds the maximum distance over which surface tension can maintain a continuous liquid connection between the two solid edges. Beyond this threshold, the gravitational force overcomes the surface tension retaining force, and the molten metal drains downward. As welding proceeded to the weld rear with a further widened gap, molten metal spread downward and to both weld sides under the synergistic effect of gravity, surface tension, and molten pool dynamic pressure, forming numerous collapse cavities on the weld surface. Increasing welding current slightly shifted the locations of weld collapse and cavities away from the arc start end, with gap tolerance only rising from 1.61 mm to 1.65 mm, an increase of only 2.5%. This is because higher current synchronously boosts droplet transfer frequency and heat input, increasing filler metal volume, expanding the molten pool and enhancing metal fluidity, which mitigates small-gap molten pool collapse to some extent. However, the large gap at the weld rear still led to insufficient molten metal bridging capacity, accompanied by a small number of collapse cavities. The weak current dependence of gap tolerance can be physically explained by the fact that although increased current provides more filler metal, the gap-induced loss of lateral support and the increased free surface area are the dominant factors controlling collapse. The additional filler metal cannot compensate for the fundamental change in the mechanical support condition once the gap exceeds the bridging threshold. This weak improvement with increasing current is consistent with the findings of Hao et al. [15], who reported that heat input adjustments had a limited effect on gap bridging once the gap exceeded a critical size.

**Figure 17:**
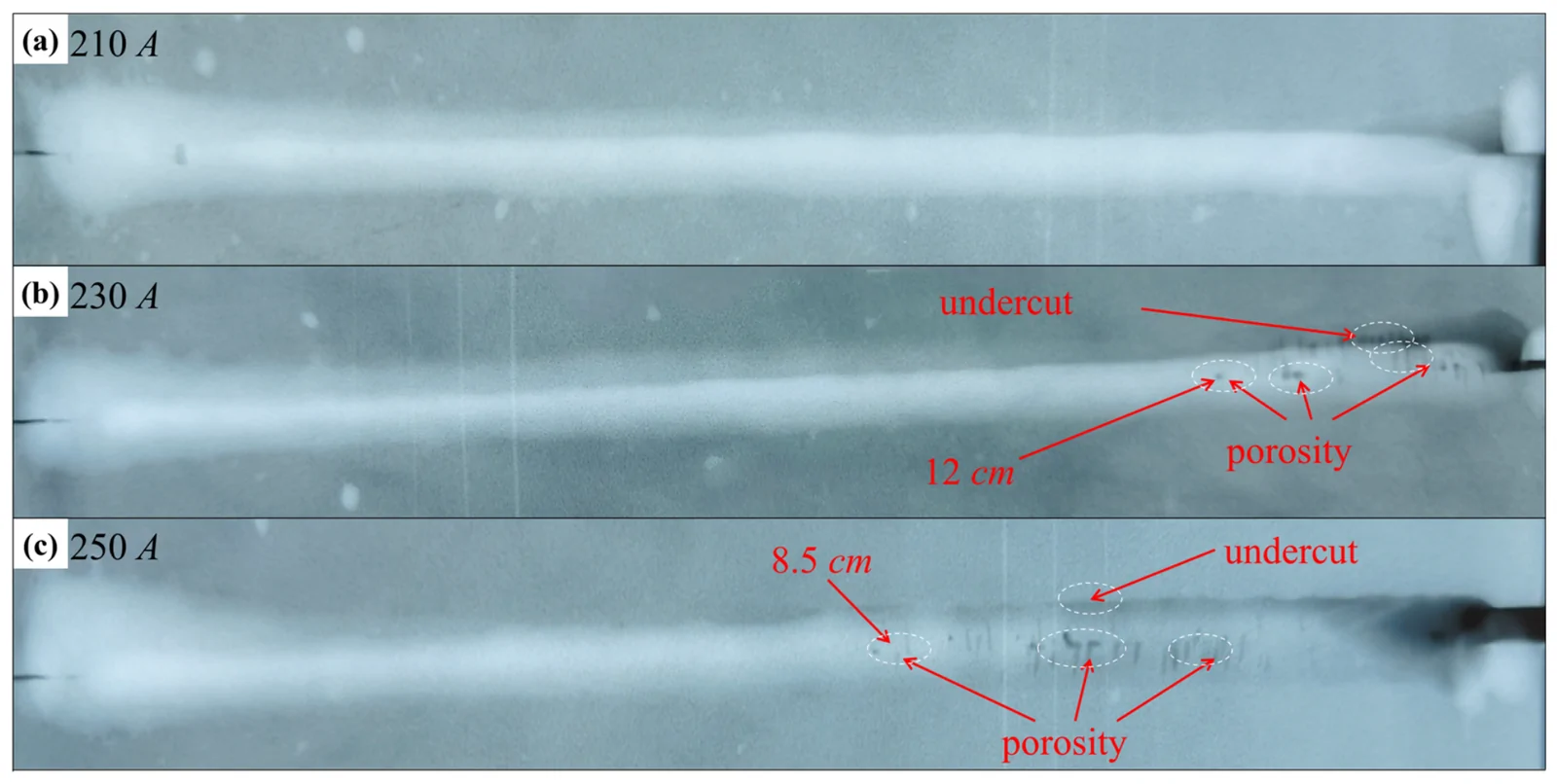
X-ray photographs of misalignment welding with different welding currents: (**a**) 210 A; (**b**) 230 A; (**c**) 250 A.

The misalignment tolerance is defined as the maximum step height (misalignment) at which the weld can maintain both acceptable surface formation and acceptable internal quality. Unlike gap tolerance, which is determined primarily by surface morphology, misalignment tolerance is governed by internal porosity formation detected by X-ray inspection. As misalignment increases, the step effect induces greater molten pool turbulence, unbalanced liquid metal flow, and delayed bubble escape, ultimately resulting in solidification porosity. The misalignment tolerance is therefore recorded as the critical misalignment at which internal porosity first becomes detectable in X-ray radiographs. Welding current significantly affects weld morphology and misalignment tolerance. Table 4 lists weld surface morphology and misalignment tolerance under different welding currents. All tested currents yielded good weld surface formation, with no obvious collapse, undercut, or other defects. As shown in Figure 17, X-ray inspection of weld internal quality shows that despite favorable surface conditions across all currents, internal quality differs remarkably. At 210 A, low heat input ensured smooth molten pool flow and low defect probability, leading to excellent weld surface formation and internal quality. At 230 A, the initial weld section was free of pores and other defects, but internal pores emerge when misalignment reached 2.57 mm. This is because small misalignment enables the heat source to fully melt the weld zone, maintaining stable molten pool flow and smooth bubble escape to avoid internal porosity. With increasing misalignment, the welded joint’s geometric profile changed, and the step effect between the assembly’s high and low sides became more prominent, causing greater molten pool turbulence, unbalanced liquid metal flow, and delayed bubble discharge, which ultimately resulted in solidification porosity. At 250 A, synchronous rises in arc heat input and droplet transfer frequency complicated internal molten pool flow, leading to weld porosity at a misalignment of only 1.82 mm. This indicates that intense molten pool flow and rapid droplet transfer further reduces weld tolerance for misalignment deviations. The negative correlation between welding current and misalignment tolerance is physically because higher current intensifies the droplet impact momentum and the electromagnetic stirring effect, which, under misalignment conditions, amplifies the flow disturbance caused by the step geometry. The more turbulent flow entrains more gas and generates more vigorous keyhole fluctuations, while the faster solidification at higher current may trap bubbles before they can escape. This phenomenon is consistent with the findings of Jing et al. [14] in narrow-gap laser welding, where increased heat input promoted melt flow instability and hot cracking when geometric asymmetry was present.

## 5. Conclusions

In this study, a transient numerical model is developed to simulate molten pool dynamics during laser–MIG hybrid welding of 10CrNiCu steel under various assembly conditions, incorporating the thermomechanical coupling among droplets, keyholes, and the molten pool. The influence of gap size and misalignment on heat and mass transfer as well as fluid flow within the molten pool is examined in detail. The main findings are summarized as follows.

(1) As the gap size increases from 0 to 1.6 mm, the peak flow velocity in the molten pool drops from approximately 1.5 m/s to 0.28 m/s, a reduction of 81.3%. The heat distribution range broadens; the keyhole depth decreases, and the pool boundary changes from a goblet shape to a nearly linear contour. At a gap of 0.4 mm, the heat input remains concentrated, and the pool morphology resembles that under the gap-free condition. At 1.6 mm, however, the reduced heat transfer efficiency delays solidification, and the gentle flow with a peak velocity of only 0.28 m/s significantly promotes undercut and lack-of-fusion defects.
(2) At small misalignments not exceeding 0.8 mm, the liquid metal spreads symmetrically under the balance of vapor recoil pressure and surface tension. When the misalignment increases to 2.8 mm, the step effect intensifies. The transverse migration velocity from the high side to the low side decreases from 0.83 m/s to 0.48 m/s, a drop of 42%. Gravity drives a net downward flow on the low side, resulting in severe pool asymmetry. Concurrently, the keyhole depth increases, the keyhole wall becomes unstable, and the risk of porosity defects rises significantly. X-ray inspection reveals that when misalignment reaches 2.57 mm, internal pores emerge massively, whereas below this threshold, the weld remains defect-free.
(3) Both assembly gaps and misalignments alter the flow patterns and heat distribution characteristics of the molten pool in laser–MIG hybrid welding, and the mechanisms by which the two affect molten pool behavior differ. The gap influences the molten pool morphology and filling effect primarily by changing the flow space and resistance characteristics of liquid metal, while the misalignment leads to the asymmetric formation of the molten pool by inducing the directional flow of liquid metal and the uneven distribution of heat input.
(4) The welding current has only a marginal effect on gap tolerance. Raising the current from 210 A to 250 A increases the tolerable gap from 1.61 mm to 1.65 mm, an improvement of only 2.5%. In contrast, for misalignment tolerance, higher currents are detrimental. The tolerable misalignment drops from 3.00 mm at 210 A to 1.82 mm at 250 A, a reduction of 39.3%. This is because the intensified droplet impact and electromagnetic stirring at higher currents exacerbate flow turbulence and keyhole fluctuations, promoting earlier porosity formation. Specifically, at 250 A, pores appear when the misalignment reaches only 1.82 mm, whereas at 210 A, they appear only when the misalignment exceeds 3.00 mm.

## Figures and Tables

**Table 1:** Chemical compositions of the 10CrNiCu metal (wt.%).

C	Si	Mn	P	S	Cu	Ni	Cr	Fe
≤0.11	0.35–0.80	0.60–1.20	≤0.025	≤0.015	0.60–0.90	0.50–0.80	0.60–0.90	Bal

**Table 2:** Thermo-physical properties of material and other coefficients used in calculation.

Physical Quantity	Value
Density *ρ* (kg/m^3^)	7600
Coefficient of thermal expansion *β*_0_ (K)	3.54 × 10^−5^
Surface tension coefficient *γ* (N/m)	1.3
Temperature coefficient of surface tension $\frac{\partial \gamma }{\partial T}$ (N∙m^−1^∙K^−1^)	−4.7 × 10^−4^
latent heat of evaporation *H_evp_* (J∙kg^−1^)	6.34 × 10^6^
Specific heat *C_p_* (J∙kg^−1^K^−1^)	*C_p_*(*T*)
Thermal conductivity *k* (Wm^−1^K^−1^)	*k*(*T*)
Permeability *μ*_0_	1.256 × 10^−6^
Latent heat of fusion *L_m_* (J/kg)	2.47 × 10^5^
liquid dynamic viscosity *μ* (kgm^−3^s^−1^)	0.007
Boltzmann constant *k_B_* (J/K)	1.38 × 10^−23^
Convective heat transfer coefficient *h_c_* (Wm^−1^K^−1^)	80
Surface emissivity (*ε*)	0.4
Avogadro constant *N_a_*	6.022 × 10^23^
Ambient temperature *T*_0_ (K)	300
Liquidus temperature *T_l_* (K)	1727
Solidus temperature *T_s_* (K)	1670
Stefan–Boltzmann constant *σ* (W/(m^2^·K^4^))	5.67 × 10^−8^

**Table 3:** Weld morphology and gap tolerance of 10CrNiCu steel with different welding current.

Current/A		Weld Morphology	Gap Tolerance (mm)
210 A	Front	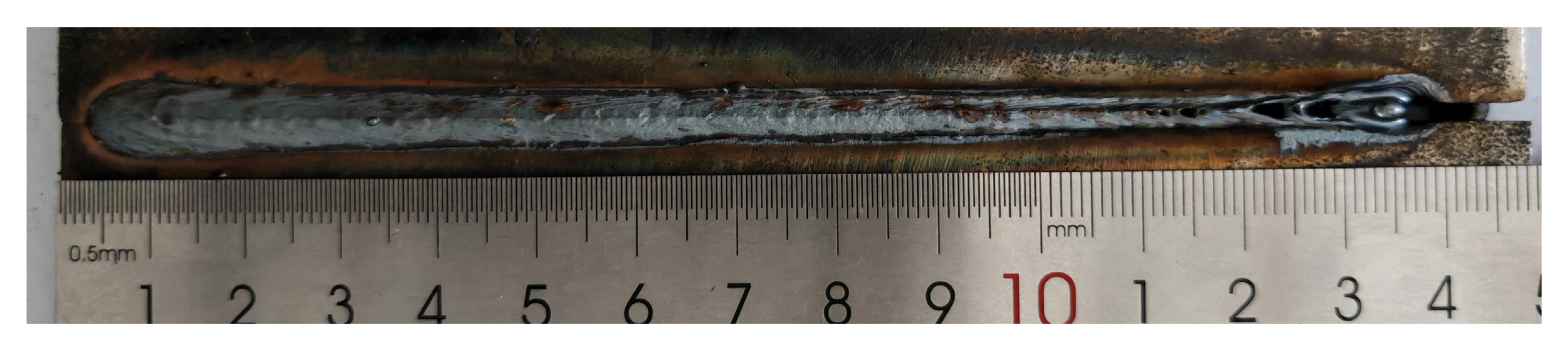	1.61 ± 0.01 mm
	Back	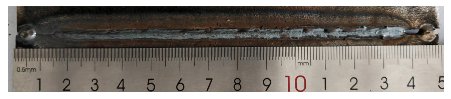	
230 A	Front	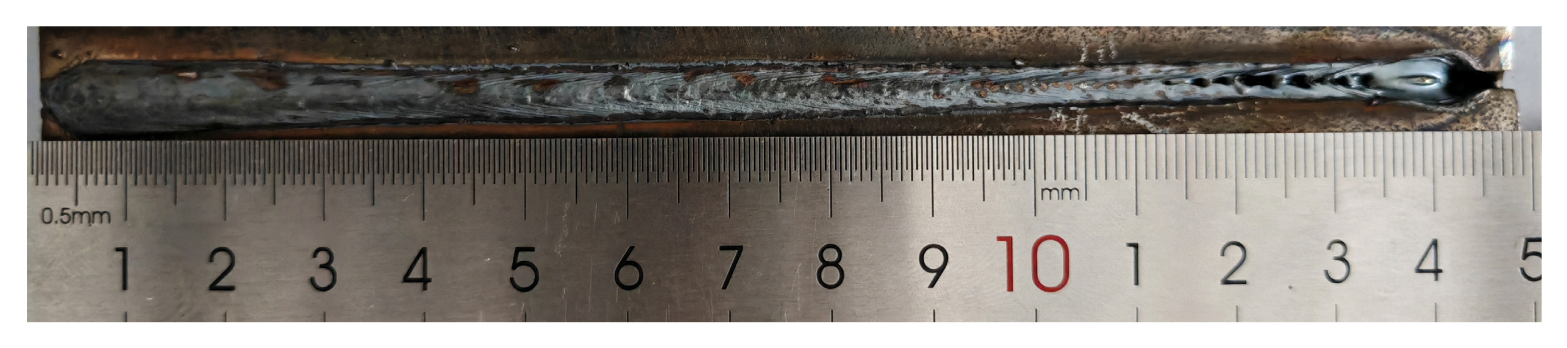	1.63 ± 0.01 mm
	Back	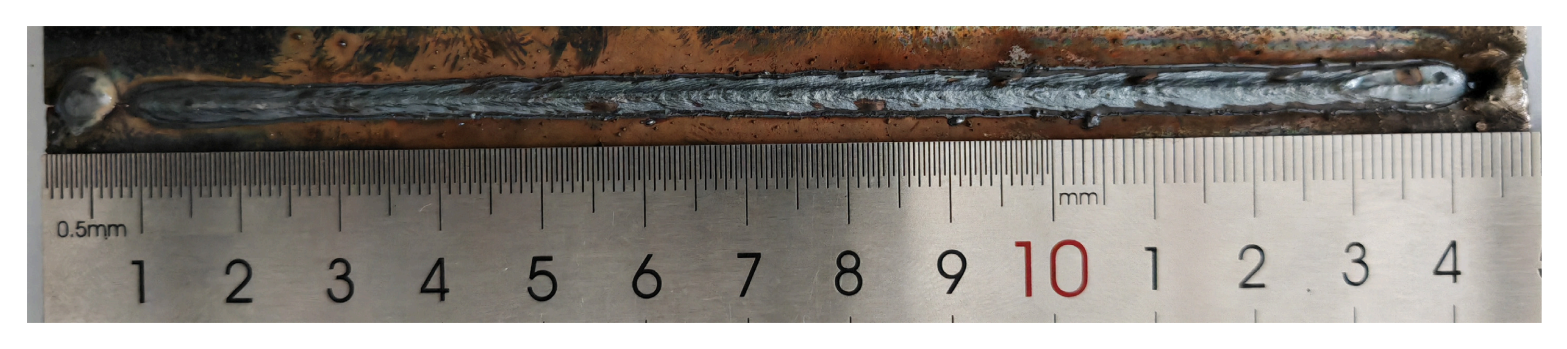	
250 A	Front	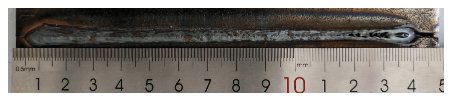	1.65 ± 0.01 mm
	Back	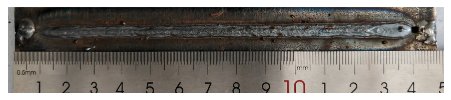	

**Table 4:** Weld morphology and misalignment tolerance of 10CrNiCu steel with different welding current.

Current/A		Weld Morphology	Misalignment Tolerance (mm)
210 A	Front	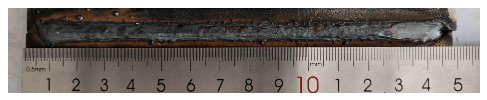	3.00 ± 0.02 mm
	Back	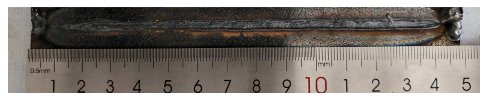	
230 A	Front	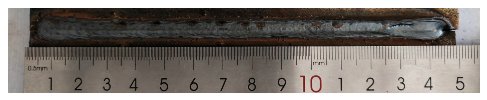	2.57 ± 0.02 mm
	Back	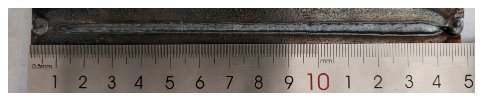	
250 A	Front	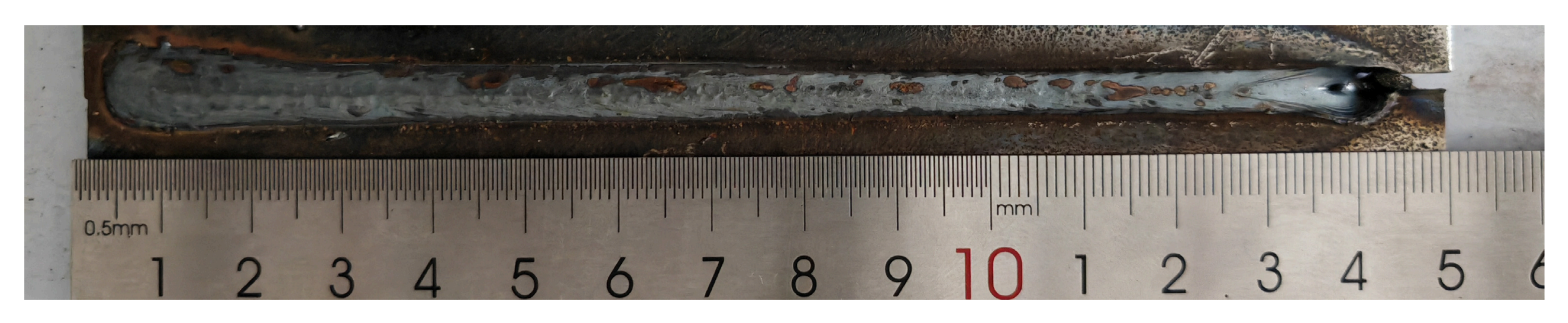	1.82 ± 0.02 mm
	Back	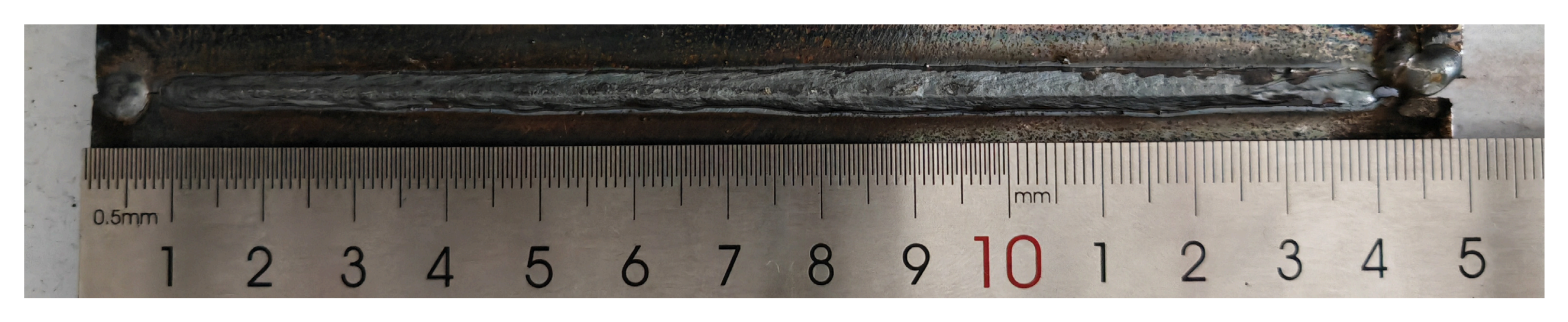	

## Data Availability

The original contributions presented in this study are included in the article. Further inquiries can be directed to the corresponding authors.
